# Exclusion of the Unfolded Protein Response in Light-Induced Retinal Degeneration in the Canine T4R *RHO* Model of Autosomal Dominant Retinitis Pigmentosa

**DOI:** 10.1371/journal.pone.0115723

**Published:** 2015-02-19

**Authors:** Stefania Marsili, Sem Genini, Raghavi Sudharsan, Jeremy Gingrich, Gustavo D. Aguirre, William A. Beltran

**Affiliations:** Section of Ophthalmology, Department of Clinical Studies, School of Veterinary Medicine, University of Pennsylvania, Philadelphia, Pennsylvania, 19104, United States of America; Univeristy of Miami, UNITED STATES

## Abstract

**Purpose:**

To examine the occurrence of endoplasmic reticulum (ER) stress and the unfolded protein response (UPR) following acute light damage in the naturally-occurring canine model of *RHO*-adRP (T4R *RHO* dog).

**Methods:**

The left eyes of T4R *RHO* dogs were briefly light-exposed and retinas collected 3, 6 and 24 hours later. The contra-lateral eyes were shielded and used as controls. To evaluate the time course of cell death, histology and TUNEL assays were performed. Electron microscopy was used to examine ultrastructural alterations in photoreceptors at 15 min, 1 hour, and 6 hours after light exposure. Gene expression of markers of ER stress and UPR were assessed by RT-PCR, qRT-PCR and western blot at the 6 hour time-point. Calpain and caspase-3 activation were assessed at 1, 3 and 6 hours after exposure.

**Results:**

A brief exposure to clinically-relevant levels of white light causes within minutes acute disruption of the rod outer segment disc membranes, followed by prominent ultrastructural alterations in the inner segments and the initiation of cell death by 6 hours. Activation of the PERK and IRE1 pathways, and downstream targets (BIP, CHOP) of the UPR was not observed. However increased transcription of caspase-12 and hsp70 occurred, as well as calpain activation, but not that of caspase-3.

**Conclusion:**

The UPR is not activated in the early phase of light-induced photoreceptor cell death in the T4R *RHO* model. Instead, disruption in rods of disc and plasma membranes within minutes after light exposure followed by increase in calpain activity and caspase-12 expression suggests a different mechanism of degeneration.

## Introduction

Retinitis pigmentosa (RP) is a clinically heterogeneous group of inherited retinal degenerative diseases leading to dysfunction and progressive loss of photoreceptor cells characterized by night vision deficits with reduction of peripheral visual field that ultimately evolves into central vision loss [1]. Presently, over 60 genes harboring mutations responsible for RP have been identified [2] (RetNet, http://www.sph.uth.tmc.edu/RetNet/); the primary defect can either occur in the retinal pigment epithelium (RPE) or in rods, with cones typically becoming involved secondarily.

Rhodopsin is the seven trans-membrane G-protein coupled receptor that, together with 11-cis retinal makes up the light-sensing protein of vertebrate rods. Rhodopsin *(RHO)* was the first gene identified as being causally-associated with RP, and since then more than 140 *RHO* mutations have been reported (http://www.hgmd.org/). Most of them are inherited in a dominant manner and account for up to 30% of autosomal dominant RP (adRP) [3–6]. In man, mutations have been described in all three domains of the protein: intradiscal, transmembrane and cytoplasmic [7]. For some of these mutations, biochemical and clinical classifications have been proposed based on *in vitro* characterization [7–11] and *in vivo* studies in patients [12].

An association between light exposure and the initiation or exacerbation of retinal degeneration has been suggested to occur in a subset of *RHO* adRP mutations [13–16], and has been experimentally demonstrated in several animal models [14,17–22]. Among them, is the T4R *RHO* mutant dog, a naturally-occurring animal model of *RHO*-adRP that shows similar phenotypic features as reported in patients with Class B1 *RHO* mutations [23]. These include a dramatically slowed time course of recovery of rod photoreceptor function after bleaching, and a distinctive topographic pattern of central retinal degeneration. The extreme sensitivity of this canine model to light has been well documented, and structural alterations have been reported to occur within minutes following acute light exposure at intensities that do not damage the wild-type (WT) retina [14,24,25]. This acute light damage results within hours in biochemical alterations [24], and within 2–4 weeks in complete loss of exposed rods, that are observed in both the tapetal (superior) and non-tapetal (inferior) regions [14,24,25].

The molecular links between *RHO* mutations and the triggering of rod cell death have been investigated, hypotheses proposed, yet the specific molecular mechanisms for most *RHO* mutations still unknown.(see for review [7]) One of the proposed mechanisms supported by both *in vitro* [8–10,26–29] and *in vivo* [29–34] studies involves misfolding of the mutant rhodopsin protein in the endoplasmic reticulum (ER) lumen as the initial trigger of ER stress, and activation of the unfolded protein response (UPR) that is mediated by three ER signal transducers: PRK-like endoplasmic reticulum kinase (PERK), inositol-requiring enzyme 1 (IRE1), and activating transcription factor 6 (ATF6). The UPR is a physiologic response to ER stress that aims at restoring ER homeostasis by inhibiting protein translation to reduce the accumulation of additional unfolded/misfolded protein; upregulating the expression of chaperones to increase the folding capacity of the ER; and activating an ER-associated degradation (ERAD) to remove unfolded/misfolded proteins from the ER membrane and deliver them to the proteasome for degradation. If ER homeostasis fails to be reestablished, some branches of the UPR may in turn activate apoptotic signals that subsequently lead to cell death (for review see [35,36]).

Although the pathogenic mechanisms of light-induced retinal degeneration in the canine T4R *RHO* model have been explored [24,25,37], the critical early molecular events that lead to the activation of photoreceptor cell death pathways have yet to be identified. In addition, the role of light as a potential trigger of an ER stress response in animal models of class B1 *RHO*-adRP has to this date not been assessed. Thus, the purpose of this study was to investigate in the naturally-occurring T4R *RHO* retinal mutant whether brief light exposure induces an ER stress and/or UPR that could be associated with the acute rod cell death.

## Materials and Methods

### Cell culture

Madin-Darby Canine Kidney Epithelial Cells [MDCK (NBL-2), ATCC CCL-34], and normal canine fibroblasts (kindly provided by Dr. Charles H Vite, University of Pennsylvania, PA) were grown in DMEM plus 10% FBS and treated with DMSO, tunicamycin (Calbiochem, EMD Chemicals, Gibbstown, NJ) at a final concentration of 2.5 μg/ml for 8 hours, or staurosporine (Sigma-Aldrich Corp, St Louis, MO) at a final concentration of 1μg/ml for 4 hours.

### Animals and light damage paradigms

Dogs were maintained at the Retinal Disease Studies (RDS) facility of the School of Veterinary Medicine, University of Pennsylvania (Kennett Square, PA). The studies were carried out in strict accordance with the recommendations in the Guide for the Care and Use of Laboratory Animals of the National Institutes of Health, the USDA’s Animal Welfare Act and Animal Welfare Regulations, and complied with the ARVO Statement for the Use of Animals in Ophthalmic and Vision Research. The protocols were approved by the Institutional Animal Care and Use Committee of the University of Pennsylvania. The dogs were part of an outbred population with a common genetic background. Six homozygous mutant (RHO ^T4R/T4R^), nine heterozygous (RHO ^T4R/+^), and four wild type (RHO ^+/+^) dogs were used. Details on the allocation of the dogs to the various experiments performed in this study are shown in Table 1. All the procedures carried out in this study, including administration of eyedrops, general anesthesia, retinal light exposure, recovery from anesthesia, euthanasia, and tissue collection, were conducted under dim red light illumination.

**Table 1:** Summary of the experimental procedures performed in the dogs of this study.

Animal ID	Genotype *RHO*	Age (weeks)	Sex	Light treatment		PE interval (hrs)	Analysis
				shielded	exposed		
***RHO* Mutant**							
EM335	T4R/+	115	M	RE	LE*	3	H&E/TUNEL assay
EM339	T4R/+	115	F	RE	LE*	6	H&E/TUNEL assay
EM340	T4R/+	115	F	RE	LE*	24	H&E/TUNEL assay
EM232	T4R/T4R	17	F	RE	LE*	0.25	TEM
EM291	T4R/+	20	F	RE	LE*	0.25	TEM
EM188	T4R/T4R	41	F	RE	LE*	1	TEM
E1051	T4R/+	17	F	RE	LE*	6	TEM
EM276	T4R/T4R	12	F	RE	LE*	6	RNA (qRT-PCR)/(RT-PCR)
EM277	T4R/T4R	12	F	RE	LE*	6	RNA (qRT-PCR)/(RT-PCR)
EM278	T4R/T4R	12	F	RE	LE*	6	RNA (qRT-PCR)/(RT-PCR)
EM279	T4R/T4R	12	F	RE	LE*	6	Western Blot (UPR & HSR)
EM267	T4R/+	12	F	RE	LE*	6	Western Blot (UPR & HSR)
EM160	T4R/+	23	F	RE	LE^◇^	1	Western Blot (Calpain study)
EM157	T4R/+	23	M	RE	LE^◇^	3	Western Blot (Calpain study)
EM156	T4R/+	23	M	RE	LE^◇^	6	Western Blot (Calpain study)
**Normal dogs**							
P1471	+/+	21	F	RE	LE*	0.25	TEM
EM262	+/+	16	F	RE	LE*	6	Western Blot (UPR & HSR)
M2367	+/+	278	F	RE	/	/	Western Blot (UPR & HSR)
M1841	+/+	172	F	RE	/	/	Western Blot (UPR & HSR)

RE: right eye; LE: left eye; H&E: Hematoxylin & Eosin histology stain; TEM: Transmission Electron Microscopy; UPR: unfolded protein response; HSR: heat shock response; qRT-PCR: quantitative real time-PCR, RT-PCR: reverse transcription PCR.

LE*: Light exposure performed using a hand-held fundus camera and taking a series of sequential overlapping retinal photographs (see methods and [26–27]).

LE^◇^: Light exposure performed using a monocular Ganzfeld and delivering a constant bright white light (6500 K, corneal irradiance: 1mW/cm^2^) for 1 min (see methods).

Following overnight dark adaptation, the dogs had the pupils of both eyes dilated with 1% tropicamide and 1% phenylephrine, 3 times, every 30 minutes. They were anesthetized with intravenous injection of ketamine (10 mg/kg) and diazepam (0.5 mg/kg), and a retrobulbar saline injection (5–10 ml) was used to prevent the ventral rotation of the globes induced by the general anesthesia, and recenter the eyes in the primary gaze. The left eyes were light-exposed (E) with either a series of sequential overlapping retinal photographs using a hand-held fundus camera (RC-2; Kowa Ltd, Nagoya, Japan) as previously described[24], or using a monocular Ganzfeld (see Table 1). The fundus camera resulted in microsecond duration flashes of a xenon lamp that produced approximate retinal doses/flash of 0.6 and 11 mJ∙cm^2^, respectively, for the tapetal and non-tapetal regions, and resulted in a >95% bleaching [23]. The monocular Ganzfeld was a component of the Espion electrophysiology system (Diagnosys LLC, Lowell, MA, USA), and delivered a constant bright white light (6500 K, corneal irradiance: 1mW/cm^2^) for 1 min. Light exposures with the fundus camera or the Espion monocular Ganzfeld do not produce any retinal damage in *WT* retinas or those affected with other inherited retinal degenerations. The contra-lateral right eyes were shielded (S) with a black photographic cloth, and served as unexposed controls. The dogs recovered from anesthesia under dim red light, and at different time-points (15 min, 1hr, 3 hrs, 6 hrs, or 24 hrs) following light exposure they were euthanized with an intravenous injection of euthanasia solution (Euthasol; Virbac, Ft. Worth, TX) and the eyes enucleated. Retinas were collected as described below.

### Histology / TUNEL assay

The eyes were fixed, trimmed and retinal cryosections were H&E stained or used for TUNEL labeling as previously reported [38].

### Quantitative real-time PCR (qRT-PCR) / Reverse Transcription PCR (RT-PCR)

The neuroretinas were collected from the eyecup under dim red light immediately after enucleation, snap-frozen in liquid nitrogen, stored at −80°C and subsequently processed for RNA studies. Total RNA from left (light exposed) and right (shielded) retinas of three homozygous mutant (*RHO* ^T4R/T4R^) dogs (Table 1) were isolated by standard TRIzol procedure (Invitrogen-Life Technologies, Carlsbad, CA), concentrations measured with a spectrophotometer (Nanodrop 1000, Thermo Fisher Scientific, Wilmington, DE), and quality verified by microcapillary electrophoresis on Agilent Bioanalyzer (Agilent, Santa Clara, CA). Only high quality (RNA A260/280 >1.8 and RIN>9) was used. RNA samples were treated with RNase-free DNase (Applied Biosystems (ABI), Foster City, CA) and 2 μg RNA was reverse-transcribed into cDNA using the High Capacity cDNA Reverse Transcriptase Kit (ABI). qRT-PCR was performed on a 7500 Real Time PCR System and software v2.0 (ABI) using 20 ng cDNA for each sample to examine the expression of 18 selected canine genes involved in ER stress: *ASK1*, *ATF4*, *BIP*, *CASP12*, *CHOP*, *DNAJA1*, *DNAJB1*, *DNAJB11*, *EDEM1 EDEM2*, *EDEM3*, *HRD1*, *HSP70*, *HSP90AA1*, *HSP90AB1*, *HSP90B1*, *VCP*, and *XBP1*. In addition, RNA levels of *CASP3* were also examined. Details on the genes are presented in Table 2 including names, descriptions and primer sequences. TaqMan reagents were used for *GAPDH* while SYBR green was used for the remaining genes. The specificity of every SYBR green assay was confirmed by dissociation curve procedures. Single melting temperatures were observed for each gene, thus excluding the presence of secondary non-specific gene products and primer dimers. For RT-PCR analysis of *XBP1* splicing, cDNA of exposed and shielded retinas of three homozygous mutants (*RHO* ^T4R/T4R^) (Table 1) was used as template for PCR amplification across the fragment of the *XBP1* cDNA bearing the unconventional intron target of IRE1α ribonuclease activity (see Table 2 for the sequences of the primers). cDNA of normal canine fibroblasts and MDCK cells that were treated with tunicamycin, dimethyl sulfoxide (DMSO), or left untreated, served as controls. PCR products were resolved on an 8% polyacrylamide/1x TBE gel.

**Table 2:** List of forward (F), reverse (R), or TaqMan expression assay (Applied Biosystems) used for qRT-PCR.

Gene	Gene description [NCBI Reference Sequence]	Sequence (5'-3') or expression assay (ABI) number
*GAPDH*	glyceraldehyde-3-phosphate dehydrogenase [NM_002046.3]	TaqMan gene expression assay: Hs02786624_g1
*ASK1*	MAP3K5 mitogen-activated protein kinase kinase kinase 5[XM_533420.5]	F: TCCCAGAGAGAGATAGCAGATAC R: CTCACTGAAAGAGCCCAGATAC
*ATF4*	activating transcription factor 4 (tax-responsive enhancer element B67), transcript variant 2 [XM_854584]	F: CGAATGGCTGGCTTTGGA R: GTCCCGGAGAAGGCATCCT
*BIP*	heat shock 70 kDa protein 5 (glucose-regulated protein, 78 kDa), transcript variant 5 (*HSPA5*) [XM_858292.2]	F: TGAAGTCACCTTTGAGATAGATGTGA R: TGTTGCCCGTACCTTTGTCTT
*CASP3*	Caspase 3 [NM_001003042.1]	F: TCGAAGCGGACTTCTTGTATG R: ACTCAAGCTTGTGAGCGTATAG
*CASP12*	Caspase 12 [NM_001077236.1]	F: GGCCGTCTGGGTGACTGAT R: ACTGCAAGGGCTGGTCACAT
*CHOP*	DNA-damage-inducible transcript 3 (DDIT3) [XM_844109.2]	F: CCCCTTGGGCCACTACCTA R: TCGTTGGCACTGGAGAAGATG
*DNAJA1*	DnaJ (Hsp40) homolog, subfamily A, member 1 [NM_001252143]	F: CTCTTGACAACCGAACCATCGT R: ACACACTTGATATCCCCATGCTT
*DNAJB1*	DnaJ (Hsp40) homolog, subfamily B, member 1, transcript variant 2 [XM_847807]	F: CCCACCCGAAAGAAGCAA R: ATAGATCTCTTCAAGCGAGACCCTAAG
*DNAJB11*	DnaJ (Hsp40) homolog, subfamily B, member 11[XM_535834.3]	F: GGAGAAGGTGAGCCTCATGTG R: ATTGGGTGCTTGACAACTTTGAT
*EDEM1*	ER degradation enhancer, mannosidase alpha-like 1[XM_533753.4]	F: GTCGGGAAGCCTGTAATGAA R: GGCATCTTCCACATCTCCTATC
*EDEM2*	ER degradation enhancer, mannosidase alpha-like 2[XM_859274.3]	F: CTTTGAGTACCTGGTGAAAGGA R: CAGTCATCGAAGCGAGTGTAA
*EDEM3*	ER degradation enhancer, mannosidase alpha-like 3[XM_537162.4]	F: GAGTAGGGAGGAGAGACAGAAG R: ATGAGTTCATCAGCTGGGTAAG
*HRD1*	synovial apoptosis inhibitor 1, synoviolin (SYVN1) [XM_540867]	F: GGCTGTGTACATGCTCTACACAGA R: CGTGTGCACCTTGATCATGAT
*HSP70*	heat shock protein 70 [NM_001003067.1]	F: GCGGAAAAGGACGAGTTTGAG R: CTGGTACAGTCCGGTGATGATG
*HSP90AA1*	heat shock protein 90kDa alpha (cytosolic), class A member 1, transcript variant 1 [XM_537557]	F: AGCTTGGGCTCGGTATCGA R: ACTCACCGCAGCACTACTATCGT
*HSP90AB1*	heat shock protein 90kDa alpha (cytosolic), class B member 1, transcript variant 1 [XM_532154]	F: AGATCACCTGGCAGTCAAGCA R: GATGAACAGCAATGCCCTGAAT
*HSP90B1*	heat shock protein 90kDa beta (Grp94), member 1 [NM_001003327]	F: TGAAAGATAAAGCTCTCAAGGACAAGA R: AGCACACGGAGACTCTGTCAGA
*VCP*	valosin containing protein[XM_847533.3]	F: CAAACGAGAGGATGAGGAAGAG R: GCCTTAAAGAGAGCAGGATGT
*XBP1total*	X-box binding protein 1 [XM_849540.2]	F: ATGGATACCCTGGCTACTGAAGAG R: CACCGGCCTCACTCCATT
*XBP1u*	X-box binding protein 1Unspliced form	F: ACTGAAGAGGAGGCGGAGAC R: GCAGAGGTGCACGTAGTCTG
*XBP1s*	X-box binding protein 1Spliced form	F: GGGATGGATACCCTGGCTAC R: CACCTGCTGCGGACTCAG
*XBP1* *	X-box binding protein 1 [XM_849540.2]	F: TTACGAGAGAAAACTCATGGCC R: GGATCCAAGTTGAACAGAATGC

Note: *GAPDH* was analyzed with TaqMan reagents, while all other genes with SYBR green.

The asterisk (*) indicates the set of primers used to detect total XBP1 (spliced and unspliced transcripts by RT-PCR.

### Statistical analysis of qRT-PCR data

All samples were run in duplicates. CT values of each gene were normalized with those of the housekeeping gene *GAPDH* and the ratio of exposed vs. shielded retinas determined with the ΔΔCT method [39]. Mean fold change (FC) differences were calculated as FC = 2^-(ΔΔCT)^. The range of FC values (FC min to FC max) were reported for each gene.Statistical significance between gene expression profiles in exposed and shielded retinas was assessed with a paired t-test.

### Protein analysis (Western Blot)

Retinal protein extracts were obtained by sonication in a buffer containing 50 mM Tris-Cl, 10 mM EGTA, 10 mM EDTA, 250 mM sucrose, 1% Triton together with a cocktail of protease inhibitors (Complete EDTA-free, Roche Applied Science, Indianapolis, IN) and phosphatase inhibitors (EMD Millipore/Calbiochem, Billerica, MA) followed by centrifugation at approximately 14,000 g for 15 min to pellet the debris. Canine fibroblasts and MDCK total cell lysates were extracted using RIPA buffer. Total protein concentration was quantified (Bradford Protein Assay, Pierce Biotechnology, Rockford, IL) and 40 μg of protein lysate for each sample was resolved on a 4–10% gradient gel and transferred to a nitrocellulose membrane (iBlot, Life Technology, Grand Island, NY). The blotted membrane was then blocked in TBST (10 mM Tris-Cl [pH 7.5], 100 mM NaCl, 0.1% Tween-20) containing 5% non-fat dry milk at room temperature for 1 hour and incubated with the specific primary antibody overnight at 4°C to detect the level of stress-induced proteins (BIP/Grp78, calnexin, GRP94, HSP70, HSP90, XBP1, eIF2α, P-elF2α, α-II spectrin, m-calpain, cleaved caspase-3). Either ß-actin or α-tubulin were used as internal controls for normalization. Details on the primary antibodies are reported in Tables 3 & 4. After washing 3x with TBST, the membrane was incubated with the appropriate secondary antibody conjugated with horseradish peroxidase (1:2,000, Zymed, San Francisco, CA) at room temperature for 1 hour. Following washing with TBST, protein signals were visualized using the ECL method according to the manufacturer's recommendations (ECL Western Blotting Detection Reagents Kit, Amersham, Piscataway, NJ), and exposed on autoradiograph films (Eastman Kodak, X-oMAT; Rochester, NY).

**Table 3:** List of primary antibodies successfully used for western blotting in the current study.

Antigen / (species)	Host	Source, Catalog No. or Name	Working Dilution
BIP/GRP78 / (human)	Rabbit mc	C.S.T.: # 3177	1:500
Calnexin / (human)	Rabbit pc	Abcam: # 13505	1:2000
Cleaved Caspase-3 (Asp175) / (human)	Rabbit pc	C.S.T.:# 9661	1:1,000
eIF2α / (human)	Rabbit pc	C.S.T.: # 9722	1:1000; 1:500
Phospho-eIF2α (ser51) / (human)	Rabbit pc	C.S.T.: # 9721	1:1000; 1:500
GRP94 / (human)	Rabbit pc	C.S.T.: # 2104	1:1000; 1:500
HSP70 / (human)	Rabbit pc	C.S.T.: # 4872	1:500
HSP70 (D69) / (human)	Rabbit pc	C.S.T.: # 4876	1:500
HSP90 / (human)	Rabbit pc	C.S.T.: # 4875	1:1000
XBP1 / (human)	Rabbit pc	A.S.B.: ARP38553_P050	1:1000
Spectrin / (chicken)	Mouse mc	EMD Millipore: MAB1622	1:2000
m-calpain (Calpain II) / (rat)	Rabbit pc	Chemicon: AB81013	1:5000
β-actin / (chicken)	Mouse mc	EMD Millipore: MAB1501	1:20000
β-actin / (human)	Mouse mc	Abcam:.# 8226	1:5000
α-tubulin / (human)	Rabbit pc	C.S.T.: # 2144	1:1000

pc: polyclonal antibody; mc: monoclonal antibody; A.S.B.: Aviva Systems Biology, San Diego, CA; C.S.T.: Cell Signaling Technology, Charlottesville, VA; S.C.T.: Santa Cruz Biotechnology, Santa Cruz, California.

**Table 4:** List of primary antibodies that were tested but failed to detect by western blotting the specific antigen in canine retina lysates when used overnight at the indicated dilution.

Antigen / (species)	Host	Source, Catalog No. or Name	Working Dilution
Activating Transcription factor 4 (ATF4) / (human, dog)	Rabbit pc	A.S.B.: # ARP38067_T100	1:1000
Activating Transcription factor 6 (ATF6) / (human)	Rabbit pc	S.C.B.: # sc-22799	1:500; 1:200
Activating Transcription factor 6 (ATF6) / (human, dog)	Rabbit pc	A.S.B.: # ARP32293_P050	1:1000; 1:500
Activating Transcription factor 6 (ATF6) / (human)	Rabbit pc	A.S.B.: # ARP31688_P050	1:750; 1:500
ASK1 / (human)	Mouse mc	Novus Biologicals: #H00004217-M03	1:500
p-ASK1 (Thr-485) / (mouse)	Rabbit pc	C.S.T.: #3765	1:1,000
Caspase-12 / (mouse)	Rabbit pc	Abcam: # ab87348	1:50
CHOP (GADD153/DDIT3) / (human)	Rabbit pc	Sigma: # G6916	1:250
CHOP (GADD153/DDIT3) / (mouse)	Rabbit pc	S.C.B.:# sc-575	1:200
CHOP (GADD153/DDIT3) / (human, pig)	Rabbit pc	A.S.B.:# ARP31591_P050	1:500

pc: polyclonal antibody; mc: monoclonal antibody; A.S.B.: Aviva Systems Biology, San Diego, CA; C.S.T.: Cell Signaling Technology, Charlottesville, VA; S.C.T.: Santa Cruz Biotechnology, Santa Cruz, California.

## Results

### Rod cell death begins 6 hours after light exposure in T4R *RHO* retinas

**Fig 1:**
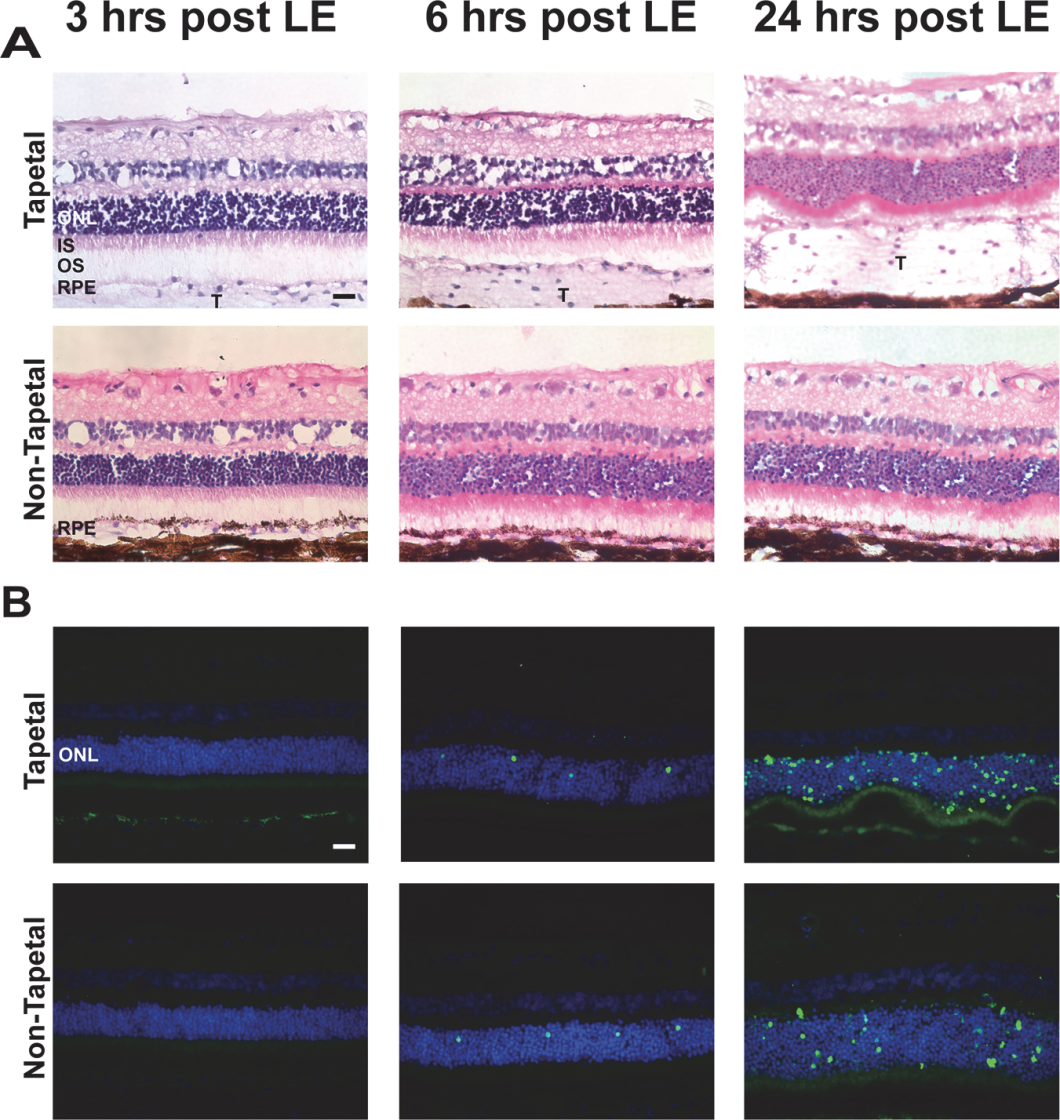
Histological alterations and photoreceptor cell death in T4R *RHO* retinas following acute light exposure. Representative photomicrographs of H&E stained retinal cryosections from RHO^T4R/+^ mutant dogs at 3, 6, and 24 hours following light exposure (LE) to a 1 min duration white light (corneal irradiance: 1mW/cm^2^). Sequential sections were used for TUNEL assay to detect the occurrence of cell death. Note that the RPE in the inferior retina is pigmented. Photomicrographs illustrate alterations seen in the tapetal /superior and non-tapetal/inferior central retina (approx. 3,000 μm from the ONH) which were first seen at 6 hours post LE and were most severe at 24 hours post LE with prominent disruption of the inner and outer segments, folding of the outer nuclear layer, and numerous features of TUNEL-positive cells. ONL: outer nuclear layer, IS; inner segments; OS; outer segments; RPE; retinal pigment epithelium; T: tapetum; scale bar = 20 μm.

At 3 hours post-exposure, there were no observable morphologic abnormalities by light microscopy on H&E stained sections from both the tapetal (superior) and non-tapetal (inferior) regions of the fundus (Fig. 1A). Earliest light microscopic changes, consisting in shortening, disorganization and fragmentation of rod outer segments, were present at the 6 hour time-point, and were more prominent at 24 hours. Consistent with these early morphological abnormalities, cell death was first detected by TUNEL labeling at 6 hours post light exposure both in the tapetal and non-tapetal regions, and was more prominent, particularly in the central retina, at 24 hours (Fig. 1B). At that time point there was greater damage in the photoreceptor layer and ONL of the tapetal than of the non-tapetal retina. This difference likely results from lack of RPE pigmentation and increased reflected light from the tapetum lucidum in the superior part of the fundus.

### Acute disruption of rod outer segment discs and inner segment organelles following light exposure in T4R *RHO* retinas

**Fig 2:**
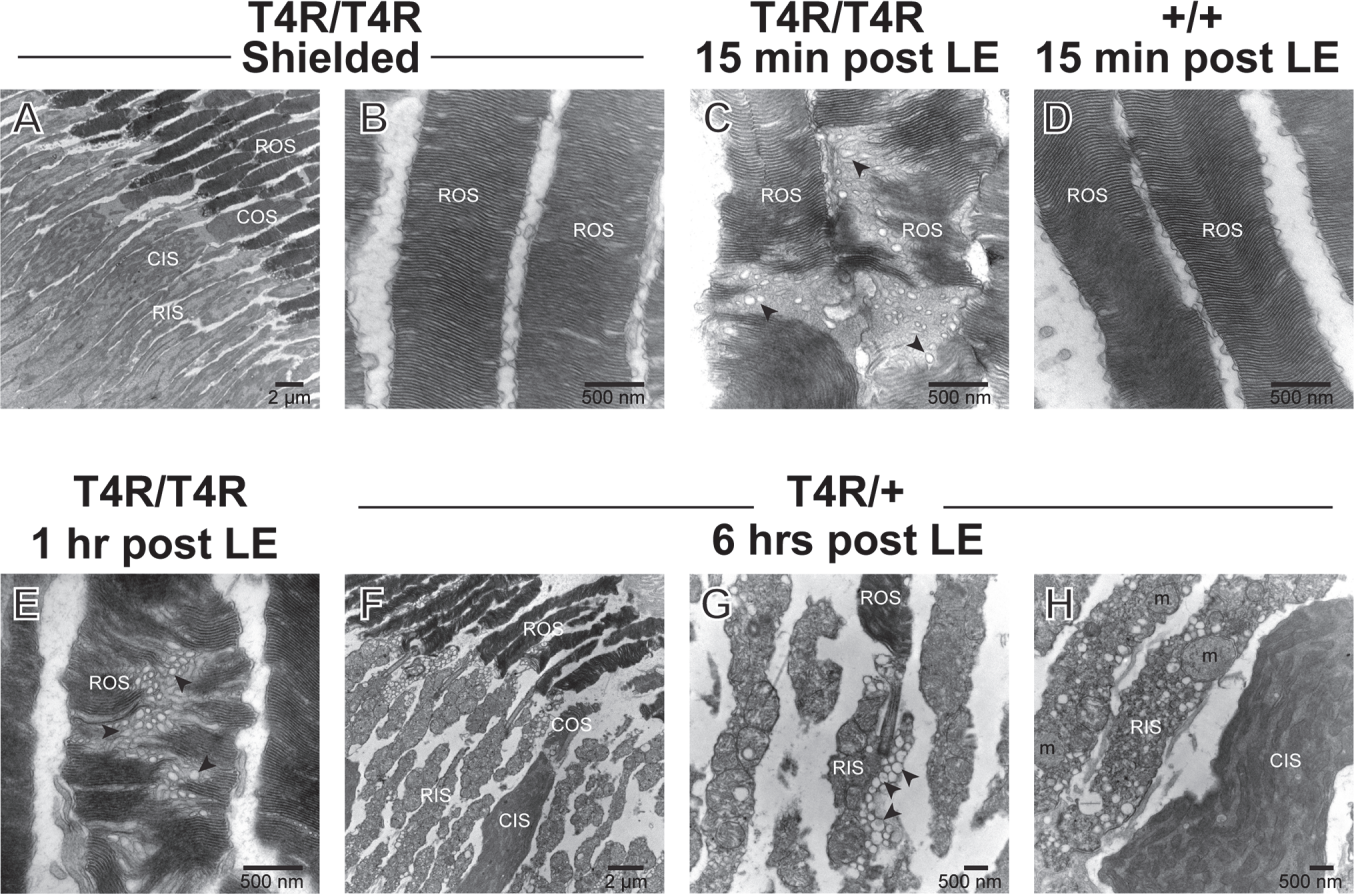
Ultrastructural alterations in rods following acute light exposure in T4R RHO canine retinas. Transmission electron micrographs of photoreceptors from T4R RHO mutant and WT canine retinas at 15 min, 1 hour, and 6 hours after light exposure (LE) to a 1 min duration of white light (corneal irradiance: 1mW/cm^2^). Black arrowheads point to vesiculo-tubular structures located in the rod outer segments (ROS) and rod inner segments (RIS) of light exposed mutant retinas. Note that the CIS and COS remain normal even though there is extensive rod degeneration. CIS; cone inner segment; m: mitochondria.

To further characterize the early stages and course of morphologic alterations that lead to the death of mutant T4R *RHO* rods following light exposure, retinas from *RHO* ^T4R/T4R^, and *RHO* ^T4R/+^ dogs were examined by transmission electron microscopy. As previously reported[24], and confirmed in this study, young *RHO* T4R mutants raised under standard kennel illumination conditions and not exposed to bright lights had normal retinal ultrastructure (Fig. 2A-B). However, as early as 15 min after bright light exposure, there was vesiculation and misalignment of rod outer segment discs in the mutants, but not in the WT retinas (compare Fig. 2C and 2D). Similar vesiculo-tubular structures were seen in ROS of mutant dogs at 1 (Fig. 2E) and 6 hours post exposure; however at this later time-point prominent alterations were also seen in the rod inner segments (RIS). These consisted in disruption of the plasma membrane, presence of single-membrane vesicles, and swelling of mitochondria (Fig. 2F-H). No such changes were seen in neighboring cones.

Based on the time course of TUNEL labeling following light exposure, and the ultrastructural studies that confirmed early structural alterations before the onset of cell death, we carried out a series of molecular and biochemical studies that focused on the ER stress response at the 6 hour post-exposure time period. This time point shows a small but significant increase in TUNEL-positive cells, an indication that cells are in the process of committing to cell death that involves many more cells by 24 hours, and continues unabated until there is extensive loss of rod photoreceptors by 2–4 weeks following exposure [14,24,25].

### Absence of ER stress and UPR activation in T4R *RHO* retinas at the onset of light-induced rod photoreceptor cell death

Although ER stress associated with retinal degeneration in some animal models of *RHO*-ADRP is likely the result of chronic accumulation of misfolded rhodopsin [29,32–34,40,41], some studies have demonstrated acute ER stress being triggered within hours following exposure to a toxic chemical [42], or to light [41,43]. This led us to examine whether the acute cell death observed at 6 hours after light exposure in the *RHO* T4R retina could be associated with disruption of ER homeostasis, and activation of an ER stress response.

**Fig 3:**
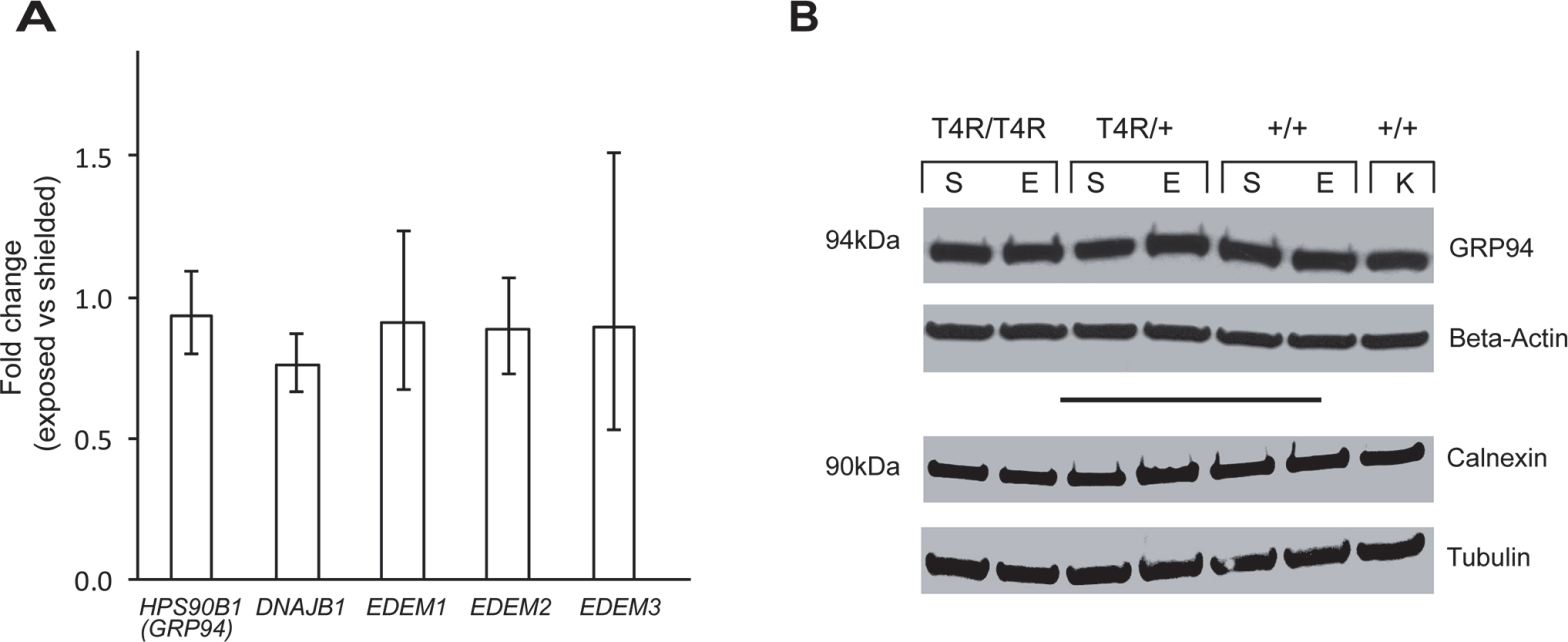
Luminal ER chaperones in T4R *RHO* and *WT* canine retinas 6 hours after light exposure. **(A)** Differential expression of genes *HSP90B1/GRP94*, *DNAJB11*, *EDEM1*, *EDEM2*, *and EDEM3* in the retinas of three RHO ^T4R/T4R^ mutant dogs following light exposure. Displayed are the mean fold change (FC) differences compared to the contralateral shielded retinas. Error bars represent the FC range (FC min to FC max). **(B)** Immunoblots showing the protein level of ER luminal chaperones GRP94 and Calnexin in light exposed (E) compared to shielded (S) retinas of mutant (RHO ^T4R/T4R^, and RHO ^T4R/+^), and wild-type RHO (+/+) dogs. A single retina from a wild-type dog kept under standard ambient kennel illumination (K) was included as a control of basal levels of GRP94, and calnexin proteins. There is no change in protein levels associated with light exposure.

We began by examining the levels of expression of intraluminal chaperones involved in the maintenance of ER homeostasis. Heat shock protein 90 kDa beta member 1 (HSP90B1, also known as GRP94, GP96, ERp99) is an ER paralog of heat shock protein 90 (HSP90) that plays a role in stabilizing and folding proteins in the ER. Like other members of the HSP family, its levels of expression are increased with the accumulation of misfolded proteins [44]. qRT-PCR analysis did not show any statistically significant changes in expression between exposed and shielded eyes of RHO ^T4R/T4R^ dogs (Fig. 3A). Similarly, no differences in protein levels were seen 6 hours following light exposure in mutant (homozygous, and heterozygous) and WT dogs (Fig. 3B). As well, no statistically significant differences were seen at the RNA level for *DNAJ* (*Hsp40*) and Homolog subfamily B member (*DNAJB11*, also known as *HEDJ*, *ERdj3*) (Fig. 3A), a soluble glycoprotein of the ER lumen that serves as a co-chaperone for BIP (also known as GRP78/HSPA5) which is the central regulator of ER stress, by stimulating its ATPase activity [45,46]. No changes were also seen in transcript levels of *EDEM1*, *EDEM2*, and *EDM3*, three ER-stress-induced members of the glycosyl hydrolase 47 family that play a role in degradation of folding defective glycoproteins [47]. In addition, western blot analysis of calnexin, an integral protein of the ER that assists in protein folding and quality control by retaining in the ER unfolded or unassembled N-linked glycoproteins [48], revealed that protein levels were not altered following light exposure in the mutant (homozygous and heterozygous) retina (Fig. 3B).

To determine whether an UPR occurred following light exposure in the T4R *RHO* mutant retina we examined the three branches of the response that can be activated following accumulation of a misfolded protein, and the subsequent dissociation of BIP from the three ER stress transducers (PERK, IRE1, and ATF6).

Activation of the PERK pathway is initiated after the dimerization and autophosphorylation of PERK which subsequently phosphorylates the eukaryotic initiation factor eIF2α. Phosphorylation of eIF2α leads to global reduction in protein synthesis to reduce ER overload. However eIF2α also can promote transcription of activating transcriptional factor 4 (ATF4), which, in turn, can increase the expression of the central ER chaperone *BIP/GRP94*. ATF4 is also known to activate the expression of apoptosis-related genes such as C/EBP-homologous protein (*CHOP*, also known as *GADD153*, *DDIT3*) (For review see [35,36,49]).

**Fig 4:**
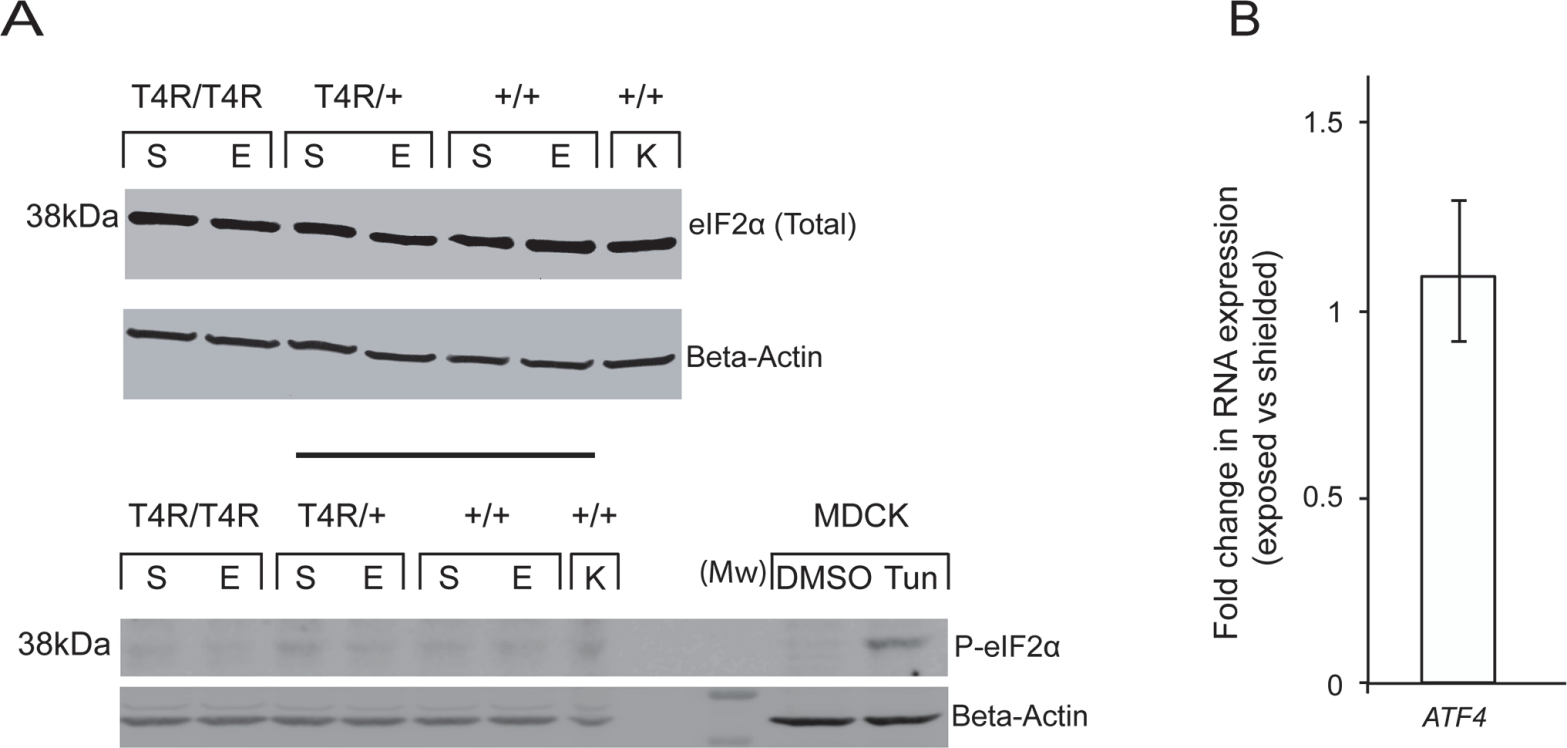
PERK-elF2α-ATF4 pathway in mutant T4R *RHO* and *WT* canine retinas 6 hours after light exposure. **(A)** Immunoblots showing the protein levels of total and phosphorylated forms of eIF2α in light exposed (E) compared to shielded (S) retinas of mutant (*RHO* ^T4R/T4R^, and *RHO* ^T4R/+^), and *WT* (+/+) dogs. A single retina from a *WT* dog kept under standard ambient kennel illumination (K) was included as a control of basal levels of total and phosphorylated eIF2α. MDCK cells either treated with DMSO or Tunicamycin (Tun) were used as controls of P-eIF2α expression and antibody specificity. **(B)** Differential expression of gene *ATF4* in the retinas of three RHO ^T4R/T4R^ mutant dogs following light exposure. Displayed is the mean fold change (FC) difference compared to the contralateral shielded retinas. Error bars represent the FC range (FC min to FC max).

Western blot analysis revealed similar levels of eIF2α in shielded and light exposed retinas from mutant (homozygous and heterozygous) T4R *RHO* and WT dogs (Fig. 4A, upper portion). A very faint band corresponding to the phosphorylated form of eIF2α was similarly detected in both exposed and shielded retinas suggesting that that there was no activation of eIF2α beyond the low basal levels. Detection of a single band at the correct molecular weight in protein extracts from MDCK cells treated with the ER-stress inducer tunicamycin confirmed the specificity of the P-eIF2α antibody against the canine amino-acid sequence (Fig. 4A, lower portion). Consistent with the absence of activation of eIF2α we did not detect by qRT-PCR any increased expression of the downstream *ATF4* transcript following light exposure (Fig 4B). The results, therefore, did not show any evidence for activation of the PERK pathway 6 hours after a light exposure that results in rod degeneration in the T4R *RHO* retina.

**Fig 5:**
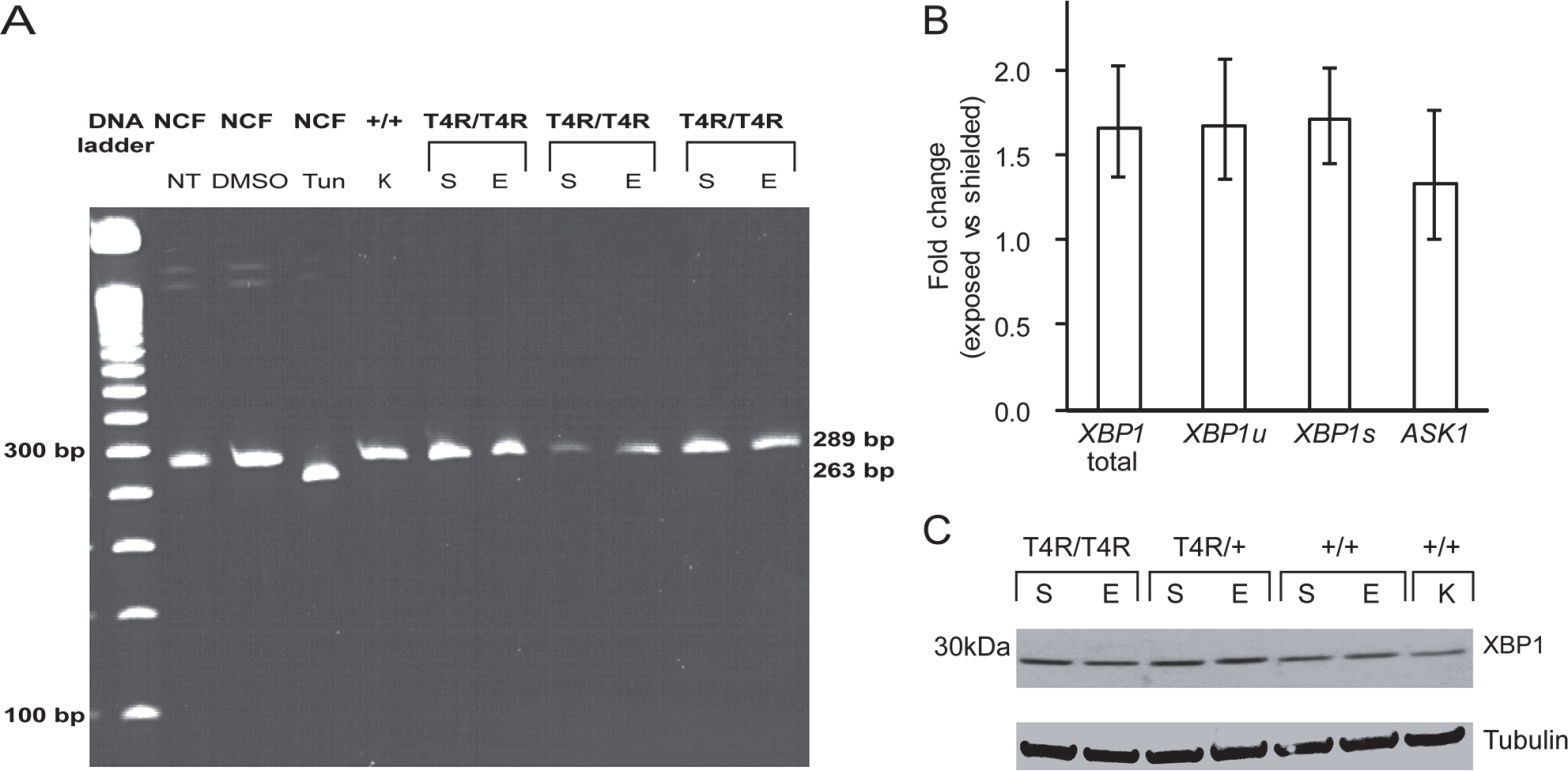
IRE1-XBP1 pathway in mutant T4R *RHO* and *WT* canine retinas 6 hours after light exposure. **(A)** RT-PCR analysis of *XBP1* splicing in light exposed (E) compared to shielded (S) T4R *RHO* and *WT* retinas. RT-PCR of canine *XBP1* generated a 289 bp fragment, which represents the unspliced form of canine *XBP1*. The 263 bp fragment, which represents the spliced form of canine *XBP1* was not observed except in the tunicamycin treated normal canine fibloblasts (NCF). A retina from a wild-type dog kept under standard ambient kennel illumination (K) was used as a control of basal *XBP1* expression and splicing. **(B)** Differential expression of genes *XBP1 and ASK1* in the retinas of three RHO ^T4R/T4R^ mutant dogs following light exposure. Three different sets of primers were used to specifically amplify the unspliced (u), spliced (s) and both (total) *XBP1* transcripts. Displayed are the mean fold change (FC) difference compared to the contralateral shielded retinas. Error bars represent the FC range (FC min to FC max). **(C)** Immunoblots showing the protein levels of total and phosphorylated forms of XBP1 in light exposed (E) compared to shielded (S) retinas of mutant (*RHO* ^T4R/T4R^, and *RHO* ^T4R/+^), and *WT* (+/+) dogs. A single retina from a wild-type dog kept under standard ambient kennel illumination (K) was included as a control of basal levels of XBP1.

The IRE1 branch of the UPR is activated after oligomerization and autophosphorylation of the IRE1α sensor, a ubiquitously expressed Ser/Thr protein kinase that also harbors an endoribonuclease domain. Activated IRE1 catalyzes the unconventional splicing of the mRNA of X-box binding protein 1 (*XBP1*) which results in a shortened mRNA transcript [50] and protein that activates the transcription of ER chaperones and ERAD factors (see for review [35,36,49]). In order to evaluate the activation of IRE-1, we analyzed the unconventional *XBP1* mRNA splicing by RT-PCR. Our results showed that the unconventional *XBP1* mRNA splicing does not occur in the T4R *RHO* mutant retinas 6 hours after light exposure (Fig. 5A). This was further confirmed by qRT-PCR analysis using primers that specifically detect the unspliced and unconventionally spliced *XBP1* transcripts (Fig. 5B). In addition, there were no significant differences at the protein levels (Fig. 5C) between exposed and shielded eyes. *ASK1* transcript levels did not significantly vary either (Fig. 5B) but state of activation of the protein could not be assessed due to lack of antibodies that would recognize total and phosphorylated forms of ASK1 (Table 4). These results still suggest however that the IRE1 branch of the UPR is not activated in the light exposed T4R *RHO* mutant retina. In contrast, normal canine fibroblast cultures treated with the ER stress inducer tunicamycin did show unconventional splicing of *XBP1* mRNA (Fig. 5A).

The third branch of the UPR involves cleavage in the Golgi by site-1 and site-2 proteases of the activating transcription factor 6 (ATF6). The N-terminal 50 kDa fragment of ATF6 (p50ATF6) translocates to the nucleus and upregulates the expression of *BIP*, and *CHOP* (For review see [35,36,49]). Despite testing several antibodies directed against ATF6 (see Table 4) we did not identify one that recognized canine ATF6, and thus were not able to assess the cleavage of ATF6. However, downstream targets of the ATF6 pathway, BIP and CHOP, could be examined (see below), and the results indirectly rule out the activation of this branch of the UPR.

**Fig 6:**
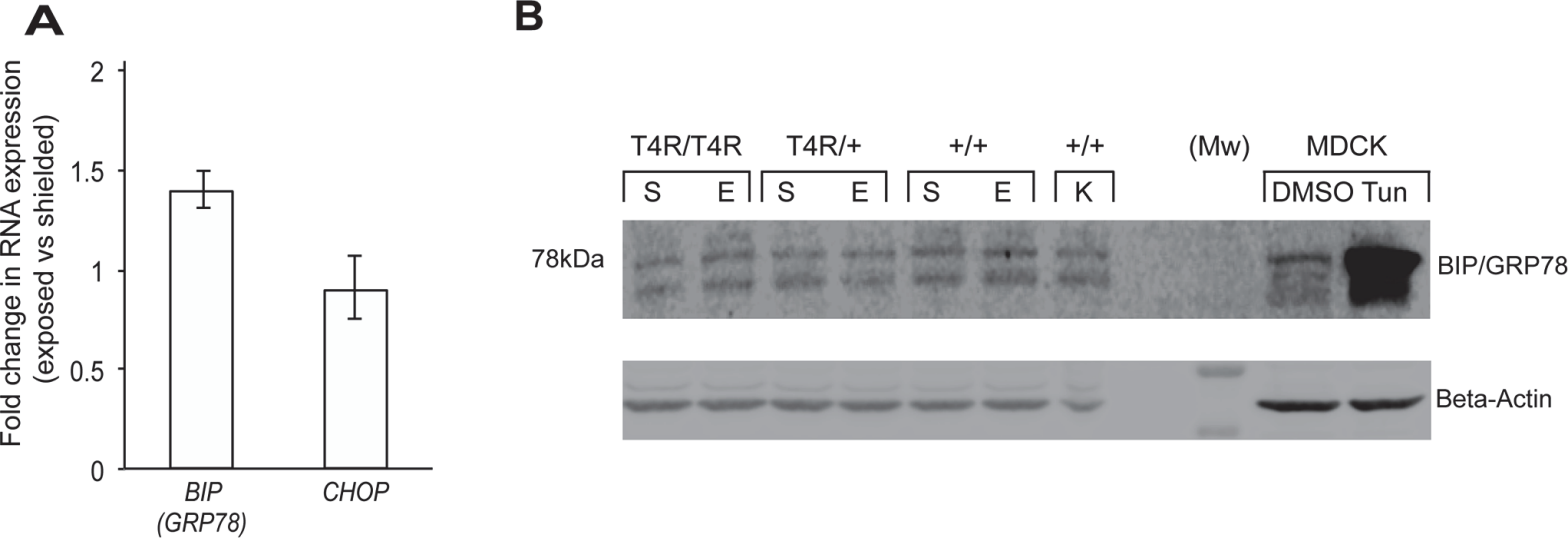
Downstream targets of the unfolded protein response (BIP and CHOP) in mutant T4R *RHO* and *WT* canine retinas 6 hours after light exposure. **(A)** Differential expression of genes *BIP/GRP78* and *CHOP* in the retinas of three RHO ^T4R/T4R^ mutant dogs following light exposure. Displayed are the mean fold change (FC) differences compared to the contralateral shielded retinas. Error bars represent the FC range (FC min to FC max). (**B)** immunoblots showing the protein levels of BIP/GRP78 in light exposed (E) compared to shielded (S) retinas of mutant (*RHO* ^T4R/T4R^, and *RHO* ^T4R/+^), and *WT* (+/+) dogs. A single retina from a wild-type dog kept under standard ambient kennel illumination (K) was included as a control of basal levels of BIP/GRP78. MDCK cells either treated with DMSO or Tunicamycin (Tun) were used as controls of BIP/GRP78 expression and antibody specificity; tunicamycin results in increased levels of BIP/GRP78.

We analyzed the expression of *BIP/GRP78* and *CHOP*, two target genes of the three branches of the UPR. BIP/GRP78 is a key chaperone induced by UPR signaling. It is an ER luminal protein that binds to each of the transducers of ER stress and serves as a sensor of alteration of ER homeostasis. Up-regulation of BIP expression promotes protein folding and reestablishment of ER homeostasis, and increased levels have been reported in genetic and light-induced models of retinal degeneration [32,33,40,41]. CHOP, also known as Growth-Arrest and DNA damage-inducible gene 153 (GADD153), is a key mediator of ER-stress induced apoptosis, and all three branches of the UPR, either independently or cooperatively, regulate its activation. Under physiological conditions, CHOP is expressed at low levels, but expression increase significantly in the presence of severe and persistent ER stress [40]. Our results showed no significant differences in RNA expression of *BIP* and *CHOP* (Fig. 6A), and protein levels of BIP (Fig. 6B) were similar between the shielded and exposed mutant retinas (homozygous and heterozygous) 6 hours after light exposure. The levels of CHOP protein could not be evaluated as three commercially-available antibodies that were tested failed to recognize canine CHOP (Table 4).

### *HSP70* cytosolic chaperone is up-regulated in T4R *RHO* retinas after light exposure

**Fig 7:**
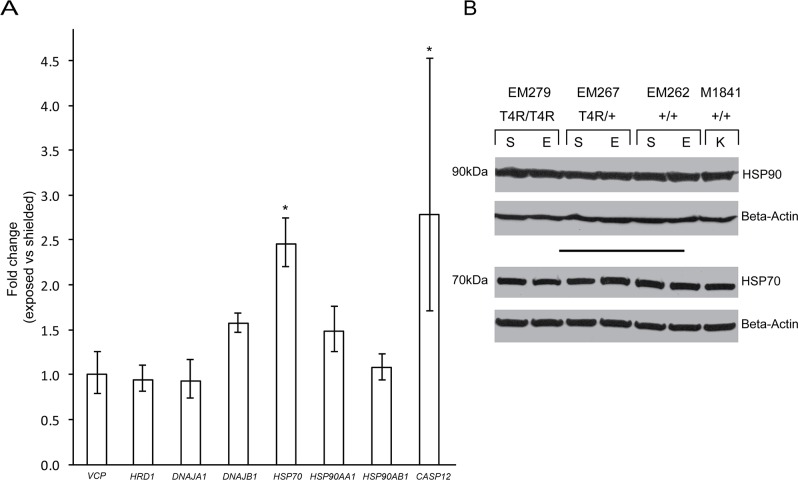
Cytosolic markers of ER associated stress and ER associated degradation (ERAD) in mutant T4R *RHO* and *WT* retinas 6 hours after light exposure. **(A)** Differential expression of genes *VCP*, *HRD1*, *DNAJA1*, *DNAJB1*, *HSP70*, *HSP90AA1*, *HSP90AB1*, *and CASP12* in the retinas of three RHO ^T4R/T4R^ mutant dogs following light exposure. Displayed are the mean fold change (FC) differences compared to the contralateral shielded retinas; error bars represent the FC range (FC min to FC max) (*: p < 0.05). (**B)** immunoblots showing the protein levels of HSP90 and HSP70 in light exposed (E) compared to shielded (S) retinas of mutant (*RHO* ^T4R/T4R^, and *RHO* ^T4R/+^), and *WT* (+/+) dogs. A single retina from a wild-type dog kept under standard ambient kennel illumination (K) was included as a control of basal levels of HSP90 and HSP70. There are no changes in HSP90 and HSP70 protein levels associated with light exposure. Note: the HSP90 antibody used recognizes the products of both the HSP90AA1 and HSP90AB1 genes.

To determine whether light exposure is associated with the activation of cytosolic chaperones that prevent misfolded protein aggregation and ultimately favor degradation via the proteasome, we examined the RNA levels in exposed and shielded mutant retinas of the following genes: *VCP* (Valosin Containing Protein), *HRD1* (ERAD-associated E3 ubiquitin protein ligase; also known as synoviolin), *DNAJA1* [DnaJ(hsp40) homolog subfamily A, member 1], *DNAJB1* [DnaJ(hsp40) homolog subfamily B, member 1], *HSP70*, *HSP90AA1* (Heat shock protein 90 kDa alpha, class A, member 1), and *HSP90AB1* (Heat shock protein 90 kDa alpha, class B, member 1). Results showed an up-regulation of *HSP70* transcription in the light-exposed retinas (p = 0.03; FC = 2.5), while no significant differences were observed for the other 7 genes (Fig. 7A). However, no differences in the protein levels of HSP70 (nor HSP90) were observed (Fig. 7B).

### Expression of ER-resident caspase-12 mRNA is up-regulated after light exposure

The involvement of ER-resident caspase-12 in retinal degeneration has been described [51–53]. To better understand the cell death mechanisms involved in photoreceptor degeneration in this large animal model, we examined the expression of the ER associated caspase-12 at the transcription level. Although there was some inter-individual variability (FC min = 1.7; FC max = 4.5), qRT-PCR analysis showed a 2.8 mean fold increase in levels of caspase-12 transcripts that was statistically significant (p = 0.025) in the light exposed mutant retinas compared to shielded controls (Fig. 7A). Absence of antibodies that recognize canine caspase-12 protein precluded confirming that it is cleaved and translocates to the nucleus.

### Evidence of calpain-activation as early as 1 hour post-light exposure in the T4R *RHO* mutant retina

**Fig 8:**
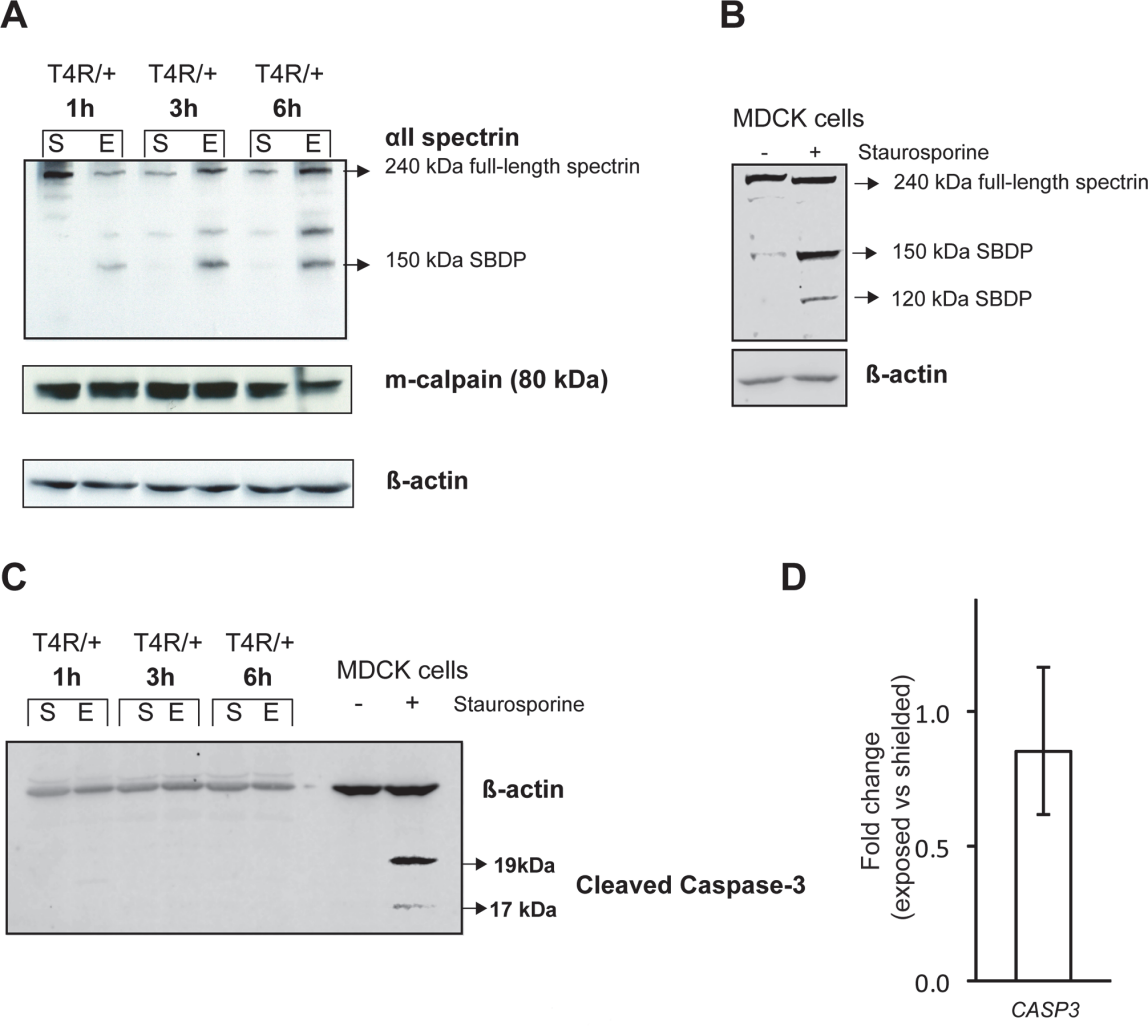
Effect of light exposure on calpain activation in mutant T4R *RHO* retinas. **(A)** Immunoblots showing the protein levels of full length and calpain-produced 150 kDa alpha II Spectrin signature breakdown product (SBDP), as well as that of m-calpain in shielded and exposed retinas of RHO ^T4R/+^ dogs at 1, 3, and 6 hours after light exposure from photographs with a Kowa RC2 fundus camera. (**B**) canine MDCK cells treated with staurosporine were used as a positive control for detection of both the calpain-cleaved (150 kDa) and caspase-3-cleaved (120 kDa) SBDPs with the antibody directed against α-II-spectrin. (C) Immunoblots showing the absence of detection of cleaved caspase-3 in shielded and exposed retinas of RHO ^T4R/+^ dogs at 1, 3, and 6 hours after light exposure. Staurosporine-treated MDCK cells were used as positive control. (**D**) Differential expression of gene *CASP3* in the retinas of three RHO ^T4R/T4R^ mutant dogs 6 hours following light exposure. Displayed are the mean fold change (FC) differences compared to the contralateral shielded retinas; error bars represent the FC range (FC min to FC max).

Accumulating evidence has been pointing out to the role of calpains in photoreceptor cell death [32,54–57]. These Ca^2+^-dependent cysteine proteases are rapidly activated following an increase in concentration of cytosolic Ca^2+^ that can be released from intracellular stores such as the ER, mitochondria, or photoreceptor discs [58]. Thus, we examined the involvement of calpain activation at 3 time points (1, 3, and 6 hours) following acute light exposure in RHO^T4R/+^ dogs by assessing alpha-II spectrin (also known as alpha-fodrin) signature breakdown products (SBDP). Both m-calpain (= calpain 1) and μ-calpain (= calpain 2) are known to induce proteolysis of alpha-II spectrin at specific sites that result in 145 and 150 kDa SBDP, while caspase 3 cleaves α-II spectrin at an additional site resulting in a 120 kDa SBDP [59,60]. Our results showed that m-calpain was expressed in both shielded and exposed retinas at all 3 time points following light exposure. α-II spectrin protein levels increased with light exposure, and a 150 kDa SBDP was found only in the exposed retinas (Fig. 8A). Absence of a 120 kDa SBDP (seen in canine MDCK cells treated with staurosporine; Fig. 8B) indicates calpain but not caspase 3 activation in the T4R *RHO* retina following acute light exposure. This was further confirmed by western blot which failed to detect any cleaved/activated caspase 3 protein in the T4R RHO retinas following light exposure (Fig 8C). No evidence of increased *CASP3* expression was either detected by qRT-PCR (Fig 8D). Thus, in the absence of results examining the occurrence of cell death at the single cell level [57], there is no evidence to suggest any involvement of Caspase 3 in this model system.

## Discussion

Transgenic animal models of *RHO*-adRP have been a common resource to investigate the cell signaling pathways that lead to photoreceptor cell death in this form of retinal degeneration. Among the mechanisms examined, the involvement of ER stress has been proposed as a common pathway in rod photoreceptor cell death in several animal models of retinal degeneration that carry different *RHO* mutations [29–33,40,41]. In this study, we examined whether ER stress, and the UPR in particular, were temporally associated with the onset of rod cell death that occurs following a short clinical light exposure in a naturally-occurring canine model of class B1 *RHO*-adRP. Our results did not identify any UPR activation concomitant with the severe ultrastructural alterations and early cell death events that occur within hours following the light exposure; instead, they point out to the extreme instability of rod disc membranes containing the mutant T4R opsin protein.

Mis-trafficking of mutant rhodopsin to the cell membrane has been shown in cultured cells [8–10,26,27,29,61,62], and in some transgenic animal models of *RHO*-adRP there is evidence of rhodopsin accumulation in rod IS [31,33,63] as well as co-localization with ER markers [30,31]. This has led several groups to hypothesize that misfolded mutant rhodopsin could induce an ER stress response. Evidence for the activation of the UPR and other ER stress markers has recently been reported in different models including: the transgenic P23H rat (lines 1 and 3) [29,34,40,41], the transgenic S334ter rat (lines 3,4 and 5) [32,41], and the T17M transgenic mouse [33].

Whether activation of the branches of the UPR reflects a compensatory mechanism to maintain ER homeostasis and promote cell survival, or on the contrary, constitutes an initial molecular event that leads to rod photoreceptor death currently is still not clear. Indeed, while increased expression of pro-apoptotic downstream targets of the UPR such as *CHOP* and *ASK1* have been reported in retinas of *RHO*-adRP models, ablation of these genes has either not modified the course of disease or negatively influenced cell survival [64,65].

**Fig 9:**
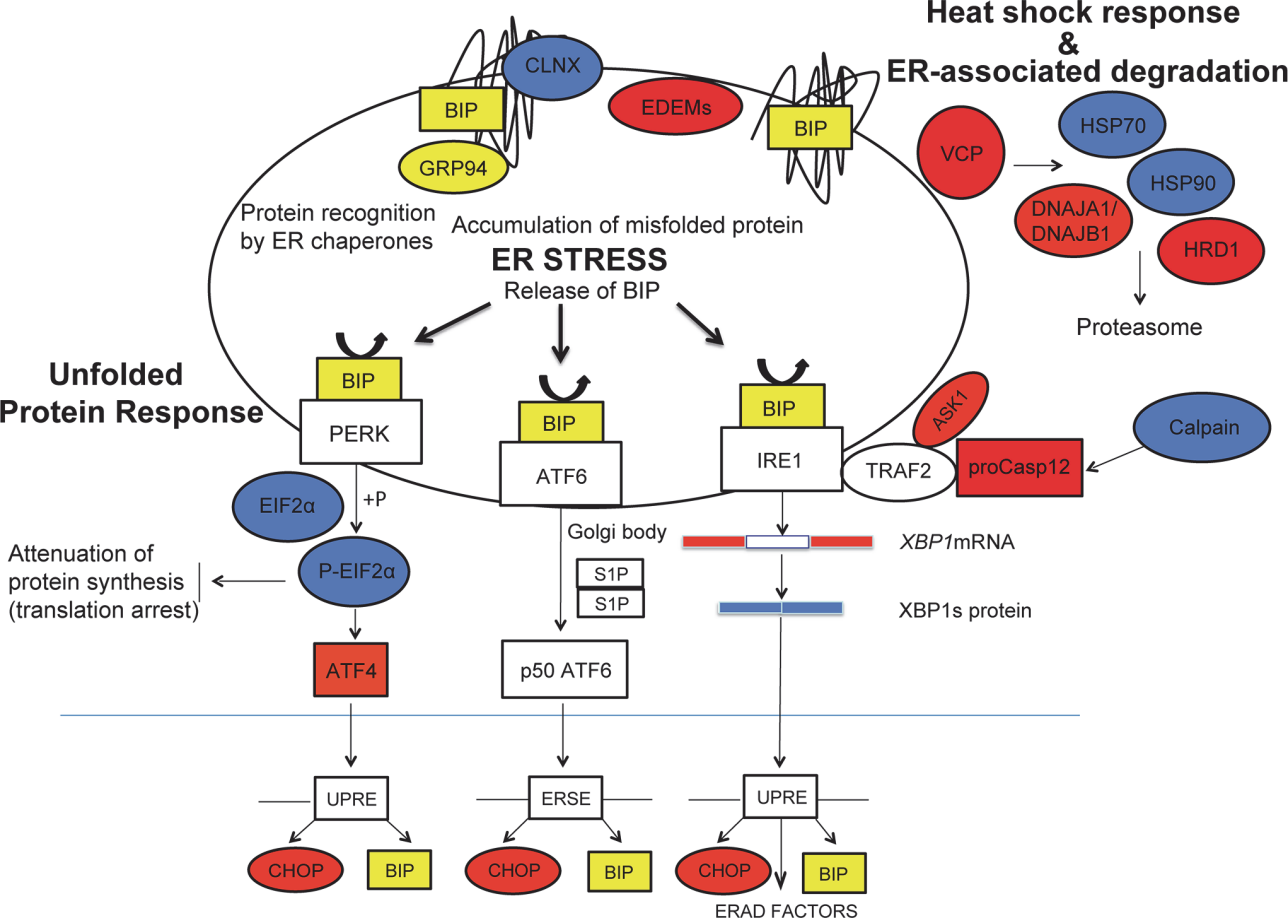
Schematic representation of the signaling pathways activated during ER stress. ER stress-related markers investigated in this study are highlighted in red (RNA), blue (protein) and yellow (both RNA and protein). (modified from [35]).

To assess the involvement of ER stress in a naturally-occurring model of *RHO*-adRP we selected the T4R *RHO* dog. Besides avoiding the increase in *RHO* gene dosage that is inherent to some transgenic animals, this model provides the opportunity to trigger a synchronized, acute rod photoreceptor degeneration following short term exposure to doses of light that are not damaging to the WT retina; the light exposures used are approximately 1000 fold or more lower in intensity than the retinal damage threshold intensities for white or medium-wavelength light in different species (see [14,24]). In this study, we detected TUNEL-labeled rods as early as 6 hours post exposure both in the tapetal and non-tapetal fundi, and by 24 hours extensive cell death was present, particularly in the central retina. Thus, to identify the early cell signalling events that are initiated following light exposure in the *RHO-*T4R retina, and that ultimately lead to cell death commitment by rods, we focused on the 6 hour time point as the majority of the photoreceptors had not yet undergone DNA cleavage and fragmentation. The analysis of the expression profile of ER markers involved in the three branches of the UPR (Fig. 9) indicates: a) the absence of chronic ER stress in the unexposed/shielded mutant retina, and b) that these pathways are not activated in the acute light-induced death of rods. During ER stress, the three associated UPR signaling pathways, PERK, IRE1 and ATF6, are typically activated [35,36,49]. In the present study only two UPR signaling pathways were examined directly, the PERK and the IREI branches. The third signaling pathway, the ATF6 branch, was not investigated due to lack of antibodies that recognize canine p50ATF6. However, we are confident that ATF6 pathway was not activated as we did not see any up-regulation of the two downstream targets: BIP and CHOP.

Rhodopsin in the T4R *RHO* mutant retina is located in rod OS and by immunohistochemistry is not retained in the ER nor aggregates in the IS [23,37]. The absence of a UPR further supports the claim that neither the lack of glycosylation at Asn(2) [37], nor the T4R mutation cause accumulation in the ER or impaired trafficking to the OS. These results resemble those recently reported for the P23H-opsin knock in mouse [66], and for the T4K and T17M transgenic Xenopus laevis where mutant RHO protein was not retained in the ER and localized normally to the rod OS [67]. The discrepancy between these findings, and that reported in P23H transgenic animals where opsin is found to accumulate in the ER, may be explained by the expression of higher levels of opsin mRNA in the transgenic models. This leads to question whether the reported occurrence of ER stress in transgenic *RHO*-adRP animals is a combination of the mutation and an increased gene dosage effect, rather than strictly the effect of the *RHO* mutation in photoreceptors. Recent evidence for an absence of increased BIP expression in rods of the T4K transgenic X. laevis following light-exposure [67] also calls for further investigation of the mechanism of action of other *RHO* mutations.

Besides activating pro-apoptotic downstream targets of the UPR such as *CHOP* and *ASK1*, ER stress can induce other signaling pathways that lead to cell death. Among them is the activation of the ER-associated caspase-12 which was found to be overexpressed in the light exposed T4R *RHO* retina. Different mechanisms for caspase-12 activation have been proposed. Pro-caspase-12 which is located on the cytoplasmic side of the ER membrane has been reported to interact with IRE1 through the adaptor molecule TRAF2 [68]. Upon ER stress, pro-caspase-12 can be released from TRAF2 to translocate from the ER to the cytosol where it directly cleaves pro-caspase-9, which in turn activates the effector caspase, caspase-3. Another proposed mechanism for pro-caspase-12 activation is via calpain cleavage [69], a pathway that has been identified in the *rd1* mouse [53]. In our study, we observed in the T4R *RHO* retina an increase in calpain activation as early as one hour after light exposure, suggesting a rapid increase in cytosolic concentrations of Ca^2+^. What are then the possible sources for such a raise in calcium levels?

Electron microscopy analysis of T4R *RHO* retinas showed prominent disruption of rod OS discs and plasma membrane as early as 15 min after a one minute period of light exposure. As the intradiscal and extracellular environments have higher concentrations of Ca^2+^ than the cytosol [58], disruption of these compartments could, within minutes, alter the intracellular calcium homeostasis. At 6 hours post light exposure there also were severe ultrastructural alterations in the rod IS with numerous single-membrane vacuoles and dilated mitochondria. Similar morphologic features have been observed in cells undergoing ER stress, where the ER swells and ribosomes dissociate from the rough ER [70,71]. As both the ER and mitochondria are major intracellular stores of Ca^2+^, loss of their membrane integrity could further contribute to the raise in cytosolic calcium. Based on our results that exclude an ER stress response as the initiating cause for the cell death process, we posit that an increase in the concentrations of cytosolic Ca^2+^ via its release from the rod intradiscal space and/or extracellular space through disruptions in the cell membranes shortly after the light exposure could subsequently affect adversely the mitochondria, and initiate the cascade of events that culminate in rod cell death.

A critical question that remains to be answered is how photobleaching of mutant T4R opsin with intensities of white light (corneal irradiance of 1mW/cm^2^: equivalent to ~1,500 lux) and exposure durations that are not toxic to the WT retina leads to the severe disruption of discal and plasma membranes. The T4R mutation which is located in the intradiscal domain affects the chromophore-binding site causing it to release the chromophore faster than WT opsin [37]. In addition, T4R opsin alone is more toxic than T4R opsin bound to 11cis-retinal as evidenced by the much accelerated course of retinal degeneration observed in double mutant dogs that also carry the RPE65 mutation depriving them from the ability to produce the 11-cis retinal chromophore [37]. One could then speculate that in the absence of chromophore, or following intense photobleaching, a change in the conformation of mutant T4R opsin alters its mobility within the lipid bilayer of the discal and cytoplasmic membranes. Similar disruption of rod OS discs as seen in our study have been reported in models of P23H *RHO* adRP including the P23H transgenic *Xenopus laevis* [63], the VPP mouse [34,72], the P23H-3 rat [34], the P23H knock in mouse [73], and more recently in the T4K transgenic Xenopus laevis following light exposure [67]. These ultrastructural alterations in discs may be explained by the recent evidence that P23H opsin tends to aggregate in the photoreceptor discs of transgenic P23H *Xenopus laevis* [63], and in the nervous system of transgenic *C*. *elegans* [74]. Similar aggregation and impaired diffusion within the lipid bilayer [63] may lead photobleached mutant T4R opsin to disturb the membrane structure, leading it to vesiculate and ultimately break down.

In summary, this study did not show any evidence of activation of the UPR in the canine T4R *RHO* model and thus does not support modulation of ER stress sensor activation [29] as a potential therapeutic venue. Besides an allele-independent corrective gene therapy approach that combines the knockdown of mutant rhodopsin mRNA and replacement with a hardened wild-type copy, pharmacological strategies aimed at stabilizing mutant opsin with locked forms of retinoids that cannot isomerize [27], or the use of cell-membrane stabilizers [75] may be beneficial for light sensitive Class B1 *RHO*-ADRP mutations that lead to disruption of discs.

## References

[1] Hartong DT, Berson EL, Dryja TP (2006) Retinitis pigmentosa. Lancet 368: 1795–1809. 17113430 10.1016/S0140-6736(06)69740-7

[2] Daiger SP, Sullivan LS, Bowne SJ (2013) Genes and mutations causing retinitis pigmentosa. Clin Genet 84: 132–141. 10.1111/cge.12203 23701314 PMC3856531

[3] Sung CH, Davenport CM, Hennessey JC, Maumenee IH, Jacobson SG, et al (1991) Rhodopsin mutations in autosomal dominant retinitis pigmentosa. Proc Natl Acad Sci U S A 88: 6481–6485. 1862076 10.1073/pnas.88.15.6481 PMC52109

[4] Inglehearn CF, Keen TJ, Bashir R, Jay M, Fitzke F, et al (1992) A completed screen for mutations of the rhodopsin gene in a panel of patients with autosomal dominant retinitis pigmentosa. Hum Mol Genet 1: 41–45. 1301135 10.1093/hmg/1.1.41

[5] Sohocki MM, Daiger SP, Bowne SJ, Rodriquez JA, Northrup H, et al (2001) Prevalence of mutations causing retinitis pigmentosa and other inherited retinopathies. Hum Mutat 17: 42–51. 11139241 10.1002/1098-1004(2001)17:1<42::AID-HUMU5>3.0.CO;2-K PMC2585107

[6] Sullivan LS, Bowne SJ, Birch DG, Hughbanks-Wheaton D, Heckenlively JR, et al (2006) Prevalence of disease-causing mutations in families with autosomal dominant retinitis pigmentosa: a screen of known genes in 200 families. Invest Ophthalmol Vis Sci 47: 3052–3064. 16799052 10.1167/iovs.05-1443 PMC2585061

[7] Mendes HF, van der Spuy J, Chapple JP, Cheetham ME (2005) Mechanisms of cell death in rhodopsin retinitis pigmentosa: implications for therapy. Trends Mol Med 11: 177–185. 15823756 10.1016/j.molmed.2005.02.007

[8] Sung CH, Davenport CM, Nathans J (1993) Rhodopsin mutations responsible for autosomal dominant retinitis pigmentosa. Clustering of functional classes along the polypeptide chain. J Biol Chem 268: 26645–26649. 8253795

[9] Sung CH, Schneider BG, Agarwal N, Papermaster DS, Nathans J (1991) Functional heterogeneity of mutant rhodopsins responsible for autosomal dominant retinitis pigmentosa. Proc Natl Acad Sci U S A 88: 8840–8844. 1924344 10.1073/pnas.88.19.8840 PMC52606

[10] Kaushal S, Khorana HG (1994) Structure and function in rhodopsin. 7. Point mutations associated with autosomal dominant retinitis pigmentosa. Biochemistry 33: 6121–6128. 8193125 10.1021/bi00186a011

[11] Krebs MP, Holden DC, Joshi P, Clark CL 3rd, Lee AH, et al (2010) Molecular mechanisms of rhodopsin retinitis pigmentosa and the efficacy of pharmacological rescue. J Mol Biol 395: 1063–1078. 10.1016/j.jmb.2009.11.015 19913029

[12] Cideciyan AV, Hood DC, Huang Y, Banin E, Li ZY, et al (1998) Disease sequence from mutant rhodopsin allele to rod and cone photoreceptor degeneration in man. Proc Natl Acad Sci U S A 95: 7103–7108. 9618546 10.1073/pnas.95.12.7103 PMC22754

[13] Heckenlively JR, Rodriguez JA, Daiger SP (1991) Autosomal dominant sectoral retinitis pigmentosa. Two families with transversion mutation in codon 23 of rhodopsin. Arch Ophthalmol 109: 84–91. 1987955 10.1001/archopht.1991.01080010086038

[14] Cideciyan AV, Jacobson SG, Aleman TS, Gu D, Pearce-Kelling SE, et al (2005) In vivo dynamics of retinal injury and repair in the rhodopsin mutant dog model of human retinitis pigmentosa. Proc Natl Acad Sci U S A 102: 5233–5238. 15784735 10.1073/pnas.0408892102 PMC555975

[15] Iannaccone A, Man D, Waseem N, Jennings BJ, Ganapathiraju M, et al (2006) Retinitis pigmentosa associated with rhodopsin mutations: Correlation between phenotypic variability and molecular effects. Vision Res 46: 4556–4567. 17014888 10.1016/j.visres.2006.08.018

[16] Paskowitz DM, LaVail MM, Duncan JL (2006) Light and inherited retinal degeneration. Br J Ophthalmol 90: 1060–1066. 16707518 10.1136/bjo.2006.097436 PMC1857196

[17] Wang M, Lam TT, Tso MO, Naash MI (1997) Expression of a mutant opsin gene increases the susceptibility of the retina to light damage. Vis Neurosci 14: 55–62. 9057268 10.1017/s0952523800008750

[18] Organisciak DT, Darrow RM, Barsalou L, Kutty RK, Wiggert B (2003) Susceptibility to retinal light damage in transgenic rats with rhodopsin mutations. Invest Ophthalmol Vis Sci 44: 486–492. 12556372 10.1167/iovs.02-0708

[19] White DA, Fritz JJ, Hauswirth WW, Kaushal S, Lewin AS (2007) Increased sensitivity to light-induced damage in a mouse model of autosomal dominant retinal disease. Invest Ophthalmol Vis Sci 48: 1942–1951. 17460245 10.1167/iovs.06-1131

[20] Tam BM, Moritz OL (2009) The role of rhodopsin glycosylation in protein folding, trafficking, and light-sensitive retinal degeneration. J Neurosci 29: 15145–15154. 10.1523/JNEUROSCI.4259-09.2009 19955366 PMC6665958

[21] Tam BM, Qazalbash A, Lee HC, Moritz OL (2010) The dependence of retinal degeneration caused by the rhodopsin P23H mutation on light exposure and vitamin a deprivation. Invest Ophthalmol Vis Sci 51: 1327–1334. 10.1167/iovs.09-4123 19933196

[22] Budzynski E, Gross AK, McAlear SD, Peachey NS, Shukla M, et al (2010) Mutations of the opsin gene (Y102H and I307N) lead to light-induced degeneration of photoreceptors and constitutive activation of phototransduction in mice. J Biol Chem 285: 14521–14533. 10.1074/jbc.M110.112409 20207741 PMC2863193

[23] Kijas JW, Cideciyan AV, Aleman TS, Pianta MJ, Pearce-Kelling SE, et al (2002) Naturally occurring rhodopsin mutation in the dog causes retinal dysfunction and degeneration mimicking human dominant retinitis pigmentosa. Proc Natl Acad Sci U S A 99: 6328–6333. 11972042 10.1073/pnas.082714499 PMC122948

[24] Gu D, Beltran WA, Li Z, Acland GM, Aguirre GD (2007) Clinical light exposure, photoreceptor degeneration, and AP-1 activation: a cell death or cell survival signal in the rhodopsin mutant retina? Invest Ophthalmol Vis Sci 48: 4907–4918. 17962438 10.1167/iovs.07-0428 PMC2377016

[25] Gu D, Beltran WA, Pearce-Kelling S, Li Z, Acland GM, et al (2009) Steroids do not prevent photoreceptor degeneration in the light-exposed T4R rhodopsin mutant dog retina irrespective of AP-1 inhibition. Invest Ophthalmol Vis Sci 50: 3482–3494. 10.1167/iovs.08-3111 19234347 PMC2742955

[26] Noorwez SM, Malhotra R, McDowell JH, Smith KA, Krebs MP, et al (2004) Retinoids assist the cellular folding of the autosomal dominant retinitis pigmentosa opsin mutant P23H. J Biol Chem 279: 16278–16284. 14769795 10.1074/jbc.M312101200

[27] Noorwez SM, Kuksa V, Imanishi Y, Zhu L, Filipek S, et al (2003) Pharmacological chaperone-mediated in vivo folding and stabilization of the P23H-opsin mutant associated with autosomal dominant retinitis pigmentosa. J Biol Chem 278: 14442–14450. 12566452 10.1074/jbc.M300087200 PMC1361689

[28] Saliba RS, Munro PM, Luthert PJ, Cheetham ME (2002) The cellular fate of mutant rhodopsin: quality control, degradation and aggresome formation. J Cell Sci 115: 2907–2918. 12082151 10.1242/jcs.115.14.2907

[29] Gorbatyuk MS, Knox T, LaVail MM, Gorbatyuk OS, Noorwez SM, et al (2010) Restoration of visual function in P23H rhodopsin transgenic rats by gene delivery of BiP/Grp78. Proc Natl Acad Sci USA 107: 5961–5966. 10.1073/pnas.0911991107 20231467 PMC2851865

[30] Frederick JM, Krasnoperova NV, Hoffmann K, Church-Kopish J, Ruther K, et al (2001) Mutant rhodopsin transgene expression on a null background. Invest Ophthalmol Vis Sci 42: 826–833. 11222546

[31] Tam BM, Moritz OL (2006) Characterization of rhodopsin P23H-induced retinal degeneration in a Xenopus laevis model of retinitis pigmentosa. Invest Ophthalmol Vis Sci 47: 3234–3241. 16877386 10.1167/iovs.06-0213

[32] Shinde VM, Sizova OS, Lin JH, LaVail MM, Gorbatyuk MS (2012) ER stress in retinal degeneration in S334ter Rho rats. PLoS ONE 7: e33266 10.1371/journal.pone.0033266 22432009 PMC3303830

[33] Kunte MM, Choudhury S, Manheim JF, Shinde VM, Miura M, et al (2012) ER Stress Is Involved in T17M Rhodopsin-Induced Retinal Degeneration. Invest Ophthalmol Vis Sci 53: 3792–3800. 10.1167/iovs.11-9235 22589437 PMC3390184

[34] Parfitt DA, Aguila M, McCulley CH, Bevilacqua D, Mendes HF, et al (2014) The heat-shock response co-inducer arimoclomol protects against retinal degeneration in rhodopsin retinitis pigmentosa. Cell Death Dis 5: e1236 10.1038/cddis.2014.214 24853414 PMC4047904

[35] Griciuc A, Aron L, Ueffing M (2011) ER stress in retinal degeneration: a target for rational therapy? Trends Mol Med 17: 442–451. 10.1016/j.molmed.2011.04.002 21620769

[36] Zhang SX, Sanders E, Fliesler SJ, Wang JJ (2014) Endoplasmic reticulum stress and the unfolded protein responses in retinal degeneration. Exp Eye Res. 10.1016/j.exer.2014.04.015 PMC4122592 24792589

[37] Zhu L, Jang GF, Jastrzebska B, Filipek S, Pearce-Kelling SE, et al (2004) A naturally occurring mutation of the opsin gene (T4R) in dogs affects glycosylation and stability of the G protein-coupled receptor. J Biol Chem 279: 53828–53839. 15459196 10.1074/jbc.M408472200 PMC1351288

[38] Beltran WA, Hammond P, Acland GM, Aguirre GD (2006) A frameshift mutation in *RPGR* exon ORF15 causes photoreceptor degeneration and inner retina remodeling in a model of X-linked retinitis pigmentosa. Invest Ophthalmol Vis Sci 47: 1669–1681. 16565408 10.1167/iovs.05-0845

[39] Livak KJ, Schmittgen TD (2001) Analysis of relative gene expression data using real-time quantitative PCR and the 2(-Delta Delta C(T)) Method. Methods 25: 402–408. 11846609 10.1006/meth.2001.1262

[40] Lin JH, Li H, Yasumura D, Cohen HR, Zhang C, et al (2007) IRE1 signaling affects cell fate during the unfolded protein response. Science 318: 944–949. 17991856 10.1126/science.1146361 PMC3670588

[41] Kroeger H, Messah C, Ahern K, Gee J, Joseph V, et al (2012) Induction of endoplasmic reticulum stress genes, BiP and chop, in genetic and environmental models of retinal degeneration. Invest Ophthalmol Vis Sci 53: 7590–7599. 10.1167/iovs.12-10221 23074209 PMC3495601

[42] Hiramatsu N, Kasai A, Du S, Takeda M, Hayakawa K, et al (2007) Rapid, transient induction of ER stress in the liver and kidney after acute exposure to heavy metal: evidence from transgenic sensor mice. FEBS Lett 581: 2055–2059. 17475259 10.1016/j.febslet.2007.04.040

[43] Nakanishi T, Shimazawa M, Sugitani S, Kudo T, Imai S, et al (2013) Role of endoplasmic reticulum stress in light-induced photoreceptor degeneration in mice. J Neurochem 125: 111–124. 10.1111/jnc.12116 23216380

[44] Yang Y, Li Z (2005) Roles of heat shock protein gp96 in the ER quality control: redundant or unique function? Mol Cells 20: 173–182. 16267390

[45] Yu M, Haslam RH, Haslam DB (2000) HEDJ, an Hsp40 co-chaperone localized to the endoplasmic reticulum of human cells. J Biol Chem 275: 24984–24992. 10827079 10.1074/jbc.M000739200

[46] Shen Y, Hendershot LM (2005) ERdj3, a stress-inducible endoplasmic reticulum DnaJ homologue, serves as a cofactor for BiP's interactions with unfolded substrates. Mol Biol Cell 16: 40–50. 15525676 10.1091/mbc.E04-05-0434 PMC539150

[47] Olivari S, Molinari M (2007) Glycoprotein folding and the role of EDEM1, EDEM2 and EDEM3 in degradation of folding-defective glycoproteins. FEBS Lett 581: 3658–3664. 17499246 10.1016/j.febslet.2007.04.070

[48] Yamashita K, Hara-Kuge S, Ohkura T (1999) Intracellular lectins associated with N-linked glycoprotein traffic. Biochim Biophys Acta 1473: 147–160. 10580135 10.1016/s0304-4165(99)00175-0

[49] Gorbatyuk M, Gorbatyuk O (2013) Review: Retinal degeneration: Focus on the unfolded protein response. Mol Vis 19: 1985–1998. 24068865 PMC3782367

[50] Uemura A, Oku M, Mori K, Yoshida H (2009) Unconventional splicing of XBP1 mRNA occurs in the cytoplasm during the mammalian unfolded protein response. J Cell Sci 122: 2877–2886. 10.1242/jcs.040584 19622636

[51] Yang LP, Wu LM, Guo XJ, Li Y, Tso MO (2008) Endoplasmic reticulum stress is activated in light-induced retinal degeneration. J Neurosci Res 86: 910–919. 17929311 10.1002/jnr.21535

[52] Yang LP, Wu LM, Guo XJ, Tso MO (2007) Activation of endoplasmic reticulum stress in degenerating photoreceptors of the rd1 mouse. Invest Ophthalmol Vis Sci 48: 5191–5198. 17962473 10.1167/iovs.07-0512

[53] Sanges D, Comitato A, Tammaro R, Marigo V (2006) Apoptosis in retinal degeneration involves cross-talk between apoptosis-inducing factor (AIF) and caspase-12 and is blocked by calpain inhibitors. Proc Natl Acad Sci U S A 103: 17366–17371. 17088543 10.1073/pnas.0606276103 PMC1859935

[54] Paquet-Durand F, Azadi S, Hauck SM, Ueffing M, van Veen T, et al (2006) Calpain is activated in degenerating photoreceptors in the rd1 mouse. J Neurochem 96: 802–814. 16405498 10.1111/j.1471-4159.2005.03628.x

[55] Mizukoshi S, Nakazawa M, Sato K, Ozaki T, Metoki T, et al (2010) Activation of mitochondrial calpain and release of apoptosis-inducing factor from mitochondria in RCS rat retinal degeneration. Exp Eye Res 91: 353–361. 10.1016/j.exer.2010.06.004 20547152

[56] Kaur J, Mencl S, Sahaboglu A, Farinelli P, van Veen T, et al (2011) Calpain and PARP activation during photoreceptor cell death in P23H and S334ter rhodopsin mutant rats. PLoS ONE 6: e22181 10.1371/journal.pone.0022181 21765948 PMC3134478

[57] Arango-Gonzalez B, Trifunovic D, Sahaboglu A, Kranz K, Michalakis S, et al (2014) Identification of a common non-apoptotic cell death mechanism in hereditary retinal degeneration. PLoS ONE 9: e112142 10.1371/journal.pone.0112142 25392995 PMC4230983

[58] Krizaj D (2012) Calcium stores in vertebrate photoreceptors. Adv Exp Med Biol 740: 873–889. 10.1007/978-94-007-2888-2_39 22453974 PMC3370389

[59] Czogalla A, Sikorski AF (2005) Spectrin and calpain: a 'target' and a 'sniper' in the pathology of neuronal cells. Cell Mol Life Sci 62: 1–12. 15990959 10.1007/s00018-005-5097-0 PMC11139101

[60] Wang KK (2000) Calpain and caspase: can you tell the difference? Trends Neurosci 23: 20–26. 10652545 10.1016/s0166-2236(99)01536-2

[61] Li T, Sandberg MA, Pawlyk BS, Rosner B, Hayes KC, et al (1998) Effect of vitamin A supplementation on rhodopsin mutants threonine-17 → methionine and proline-347 → serine in transgenic mice and in cell cultures. Proc Natl Acad Sci U S A 95: 11933–11938. 9751768 10.1073/pnas.95.20.11933 PMC21743

[62] Chen YF, Wang IJ, Lin LL, Chen MS (2011) Examining rhodopsin retention in endoplasmic reticulum and intracellular localization in vitro and in vivo by using truncated rhodopsin fragments. J Cell Biochem 112: 520–530. 10.1002/jcb.22942 21268073

[63] Haeri M, Knox BE (2012) Rhodopsin mutant P23H destabilizes rod photoreceptor disk membranes. PLoS ONE 7: e30101 10.1371/journal.pone.0030101 22276148 PMC3261860

[64] Nashine S, Bhootada Y, Lewin AS, Gorbatyuk M (2013) Ablation of C/EBP homologous protein does not protect T17M RHO mice from retinal degeneration. PLoS ONE 8: e63205 10.1371/journal.pone.0063205 23646198 PMC3640035

[65] Adekeye A, Haeri M, Solessio E, Knox BE (2014) Ablation of the proapoptotic genes chop or Ask1 does not prevent or delay loss of visual function in a P23H transgenic mouse model of retinitis pigmentosa. PLoS ONE 9: e83871 10.1371/journal.pone.0083871 24523853 PMC3921110

[66] Sakami S, Kolesnikov AV, Kefalov VJ, Palczewski K (2014) P23H opsin knock-in mice reveal a novel step in retinal rod disc morphogenesis. Hum Mol Genet 23: 1723–1741. 10.1093/hmg/ddt561 24214395 PMC3943518

[67] Tam BM, Noorwez SM, Kaushal S, Kono M, Moritz OL (2014) Photoactivation-Induced Instability of Rhodopsin Mutants T4K and T17M in Rod Outer Segments Underlies Retinal Degeneration in X. laevis Transgenic Models of Retinitis Pigmentosa. J Neurosci 34: 13336–13348. 10.1523/JNEUROSCI.1655-14.2014 25274813 PMC4180472

[68] Yoneda T, Imaizumi K, Oono K, Yui D, Gomi F, et al (2001) Activation of caspase-12, an endoplastic reticulum (ER) resident caspase, through tumor necrosis factor receptor-associated factor 2-dependent mechanism in response to the ER stress. J Biol Chem 276: 13935–13940. 11278723 10.1074/jbc.M010677200

[69] Nakagawa T, Yuan J (2000) Cross-talk between two cysteine protease families. Activation of caspase-12 by calpain in apoptosis. J Cell Biol 150: 887–894. 10953012 10.1083/jcb.150.4.887 PMC2175271

[70] Hitomi J, Katayama T, Taniguchi M, Honda A, Imaizumi K, et al (2004) Apoptosis induced by endoplasmic reticulum stress depends on activation of caspase-3 via caspase-12. Neurosci Lett 357: 127–130. 15036591 10.1016/j.neulet.2003.12.080

[71] Kobayashi T, Tanaka K, Inoue K, Kakizuka A (2002) Functional ATPase activity of p97/valosin-containing protein (VCP) is required for the quality control of endoplasmic reticulum in neuronally differentiated mammalian PC12 cells. J Biol Chem 277: 47358–47365. 12351637 10.1074/jbc.M207783200

[72] Liu X, Wu TH, Stowe S, Matsushita A, Arikawa K, et al (1997) Defective phototransductive disk membrane morphogenesis in transgenic mice expressing opsin with a mutated N-terminal domain. J Cell Sci 110 (Pt 20): 2589–2597. 9372448 10.1242/jcs.110.20.2589

[73] Sakami S, Maeda T, Bereta G, Okano K, Golczak M, et al (2011) Probing mechanisms of photoreceptor degeneration in a new mouse model of the common form of autosomal dominant retinitis pigmentosa due to P23H opsin mutations. J Biol Chem 286: 10551–10567. 10.1074/jbc.M110.209759 21224384 PMC3060508

[74] Chen Y, Jastrzebska B, Cao P, Zhang J, Wang B, et al (2014) Inherent instability of the retinitis pigmentosa P23H mutant opsin. J Biol Chem 289: 9288–9303. 10.1074/jbc.M114.551713 24515108 PMC3979360

[75] Heier CR, Damsker JM, Yu Q, Dillingham BC, Huynh T, et al (2013) VBP15, a novel anti-inflammatory and membrane-stabilizer, improves muscular dystrophy without side effects. EMBO Mol Med 5: 1569–1585. 10.1002/emmm.201302621 24014378 PMC3799580

